# Unveiling the bacterial diversity and potential of the *Avicennia marina* ecosystem for enhancing plant resilience to saline conditions

**DOI:** 10.1186/s40793-024-00642-w

**Published:** 2024-12-04

**Authors:** Amal Khalaf Alghamdi, Sabiha Parween, Heribert Hirt, Maged M. Saad

**Affiliations:** 1 DARWIN21, Biological and Environmental Science and Engineering Division, King Abdullah University of Science and Technology (KAUST), Thuwal, 23955-6900 Saudi Arabia; 2 Department of Botany and Microbiology, College of Science, King Saud University, P.O. Box 2455, Riyadh, 11451 Saudi Arabia; 3 Max Perutz Laboratories, University of Vienna, Vienna, Austria

**Keywords:** Global food security, Salt stress tolerance, Halophilic bacteria, *Avicennia marina*, Biostimulants, And Plant-microbial interaction

## Abstract

**Background:**

*Avicennia marina* ecosystems are critical for coastal protection, water quality enhancement, and biodiversity support. These unique ecosystems thrive in extreme saline conditions and host a diverse microbiome that significantly contributes to plant resilience and growth. Global food security is increasingly threatened by crop yield losses due to abiotic stresses, including saline soils. Traditional plant breeding for salt tolerance is both costly and time-consuming. This study explores the potential of bacteria from *A. marina* to enhance plant growth under saline conditions, emphasizing their ecological significance.

**Results:**

We analyzed the microbiome of *A. marina* from the Red Sea coast using high-throughput Illumina sequencing and culture-dependent methods across various compartments (bulk soil, rhizosphere, rhizoplane, roots, and leaves). Our findings revealed distinct compartment-specific microbial communities, with Proteobacteria being the dominant phylum. Functional predictions indicated diverse microbial roles in metal uptake and plant growth promotion (PGP). Remarkably, our culture-dependent methods allowed us to recover 56% of the bacterial diversity present in the microbiome, resulting in the isolation and characterization of 256 bacterial strains. These isolates were screened for PGP traits, including salt and heat tolerance, siderophore production, and pectinase activity. Out of the 77 bacterial isolates tested, 11 demonstrated a significant ability to enhance Arabidopsis growth under salt stress.

**Conclusions:**

Our study highlights the ecological significance of mangrove microbiomes and the potential of culture collections in offering innovative solutions for ecological restoration and crop production in saline conditions. The unique collection of mangrove bacteria, particularly from the rhizosphere and endophytes, showcases significant PGP traits and stress tolerance capabilities. These findings emphasize the importance of functional traits, such as salt tolerance, in the recruitment of endophytic bacteria by plants over taxonomic affiliation. The identified bacterial strains hold potential not only for developing biofertilizers to improve crop productivity but also for ecological restoration projects aimed at rehabilitating saline-degraded lands, thereby contributing to overall ecosystem health and sustainability.

**Supplementary Information:**

The online version contains supplementary material available at 10.1186/s40793-024-00642-w.

## Introduction

The world’s population is projected to grow by over 20% to approximately 10.4 billion by 2100 [1]. However, with 2 billion people living in poverty, there is a significant need to increase crop production to provide food for the growing global population. One of the critical challenges to achieving this is challenged by increasing soil salinization, which affects approximately 900 million hectares globally and causes an estimated annual loss of 27 billion USD [2]. According to USDA estimates, about 10 million hectares are lost each year globally due to salinity and/or waterlogging [3]. The widely used approaches to avoid salinity in agriculture, including developing salt-tolerant crops and conventional breeding, are labor-intensive and time-consuming. As a result, alternative, innovative technologies are urgently needed to improve agricultural sustainability. One such promising approach is the use of plant growth-promoting bacteria (PGPB) derived from extreme environments [4, 5]. The root-associated biome has complex microbial diversity and is the prototype of the natural relationship between plants and microbes [6]. Recent studies have revealed the efficiency of PGPB as salinity and drought alleviators in plants and promoting plant growth [7–9].

Although crop plants may struggle with high salinity and flooding, mangroves thrive in the intertidal zone between land and sea. Mangrove ecosystems, particularly the root-associated microbial communities, represent a natural model of salinity resilience. Mangroves like *Avicennia marina*, which thrive in hypersaline coastal wetlands of the Red Sea, have evolved to endure extreme environmental stressors, including high salinity, waterlogging, and nutrient scarcity [10, 11] These trees employ sophisticated mechanisms such as root suberization, salt excretion through leaf glands, and osmotic regulation to mitigate the detrimental effects of salinity [12, 13]. Importantly, microbial communities in the mangrove rhizosphere play a pivotal role in these adaptations, facilitating nutrient uptake and stress mitigation through various biochemical pathways [14] [12]. In the central Red Sea, the high salinity is the primary stress in many regions of mangrove areas (Al Lith, South Jeddah, Dahban, Thuwal, Mastorah, reaching highest levels in Rabigh with 40.75%) [13].

Microbial communities in mangrove sediments play an essential role in the biogeochemical recycling of carbon, nitrogen, phosphorus, and sulphur in mangrove swamps. Previous data revealed that root exudates could serve as carbon sources and secondary metabolites as antimicrobial substances in the mangrove ecosystem [14]. Therefore, the root exudates in mangroves could profoundly influence many root-associated microbial populations, especially those inhabiting the rhizosphere [15]. Kibmrel et al. [16] studied the salt-tolerant bacterial and archaeal strains of the marine environment and constructed genomes for 44 taxa, including *Alphaproteobacteria*,* Marinobacter*, and *Roseobacter*. also has lignin-deconstruction capabilities [17]. The *Roseobacter* group is adapted to the marine environment and comprises chemotrophic bacteria often associated with eukaryotes [18]. Group members have a much higher metabolic and ecological versatility than other dominant marine bacteria [19]. Actinobacteriota, such as *Streptomyces venezuelae*, were found to augment rice growth and significantly increase tolerance to salinity by reduction of ethylene, reactive oxygen species (ROS), and Na + contents while accumulating more proline, total chlorophyll, relative water content (RWC), malondialdehyde (MDA), and K + than those of uninoculated controls [20].

Stress mitigation is succeeded through various mechanisms such as biofilm formation, extracellular polymeric substances (EPS) production, nitrogen fixation, phytohormone production, and ACC deaminase activity [21]. For instance, ACC deaminase can reduce ethylene levels in plants required for signalling, adaptation, and survival under waterlogging conditions. Reducing ethylene levels in aquatic or riparian plants may antagonistically affect plant adaptability, growth, and survival [8, 22]. Along with the bacterial community, mangrove ecosystems harbor a rich and diverse fungal community that plays a fundamental role in nutrient cycling, plant health, and ecosystem stability. These fungi are particularly well adapted to the challenging conditions of mangrove habitats, which include high salinity, waterlogging, and fluctuating oxygen levels. Among them, arbuscular mycorrhizal (AM) fungi are especially significant, forming symbiotic associations with mangrove roots. AM fungi assist in nutrient acquisition, particularly phosphorus, and enhance water uptake, which is critical for plant growth and survival in saline environments. In return, AM fungi receive carbon from the host plant, creating a mutually beneficial relationship. These fungi also contribute to the soil structure and help mangrove trees manage salt stress, making them key players in the resilience and productivity of mangrove forests [23, 24].

In the present study, we investigated the bacterial composition across different compartments of *A. marina* (bulk soil, rhizosphere, rhizoplane, and root and leaf endophytes) to understand the diversity, structure, and functional potential of these communities. By investigating these distinct compartments, the study sought to gain insights into the microbial diversity and structure, abundance, and potential interrelationships between bacterial strains within each compartment. Furthermore, the study aimed to assess the capacity of highly represented culturable bacterial strains in these compartments to contribute to plant growth and resilience in the face of salinity stress using the model plant system and crop plant. By linking our findings to the urgent global challenge of soil salinization, we highlight the potential application of mangrove-associated bacteria as biofertilizers and biostimulants for crop plants. This research not only advances our understanding of the mangrove microbiome but also provides a basis for developing sustainable, microbe-based strategies for enhancing plant growth and resilience in saline environments and contribute to the development of effective strategies for the conservation, restoration, and sustainable management of these critical ecosystems.

## Materials and methods

### Study site and experimental design

**Fig. 1:**
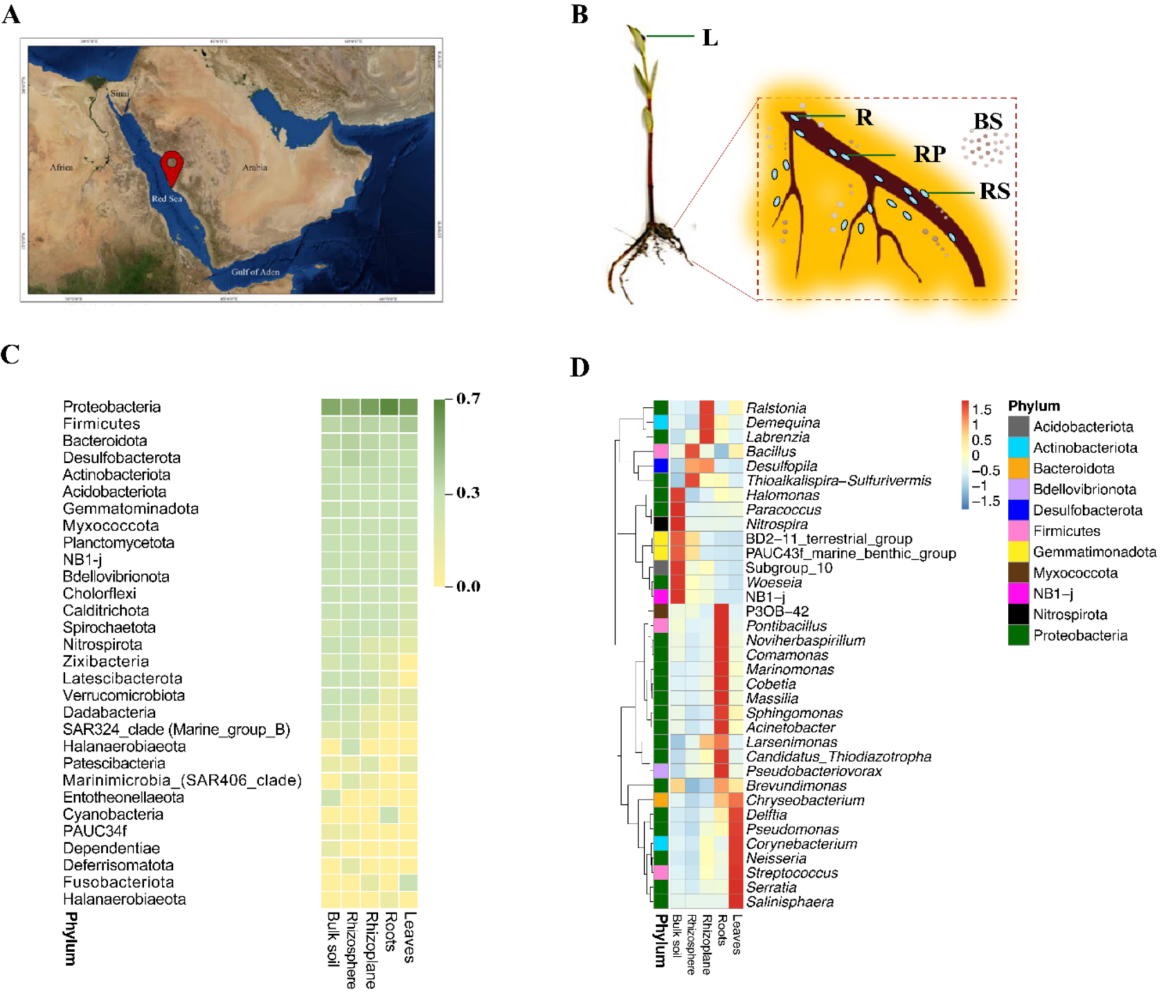
Microbial diversity and spatial distribution in *Avicennia marina* ecosystem along the red sea coast. (**A**) Geographical location of the *Avicennia marina* study site along the Red Sea coast, at KAUST, Thuwal, Saudi Arabia indicated by the red pin. (**B**) Schematic representation of the mangrove root system, illustrating key compartments: root (R), rhizoplane (RP), and rhizosphere (RS). (**C**) Heatmap depicting the relative abundance of bacterial phyla > 0.01%across different root compartments. The colour gradient indicates abundance levels, from low (light green) to high (dark green) (**D**) Functional diversity analysis of bacterial communities across compartments, shown in a color-coded grid. (**E**) Legend illustrating colour gradients used for abundance and diversity metrics

A total of 32 *A. marina* seedlings (10–30 cm) from the intertidal mud flats of KAUST, Thuwal, Saudi Arabia (22.339914°N, 39.087972°E), were collected in March 2019 (Fig. 1A). Samples were handled with sterilized tools, stored in plastic bags, and kept at 4 °C until use. The plants were separated into four compartments: rhizosphere, rhizoplane, and endosphere of the roots and leaves, with five biological replicates (*n* = 5). Roots were manually shaken to remove excess soil, then placed in sterile PBS, vortexed, and centrifuged to obtain the “Rhizosphere” fraction. Cleaned roots were sonicated in PBS for 30s to get the “Rhizoplane” fraction. Roots were surface-sterilized, sonicated in PBS again, and the remaining roots were designated as the “Roots” compartment. Leaves were surface-sterilized and designated as “Leaves”. “Bulk soil” samples were collected at a 15 cm depth from non-vegetative quadrats and sieved (pore size 2 mm) (Fig. 1B, Figures S1, and S2).

### Soil and plant sample elemental analysis

Soil pH, temperature, and salinity were measured using a Multi-Parameter Tester (C-600 A, Qolam), and dissolved oxygen with an ExtechII Dissolved Oxygen Meter (DO600, Extech). Triplicate soil and plant samples (30 g each) were dried at 60 °C for 24 h, macerated using a Mill Grinder_acl, and digested with 1 M nitric acid. Elemental analysis was performed using Inductively Coupled Plasma Optical Emission Spectrometry (ICP-OES-Agilent 5110_SW_acl), with standard solutions of elements such (Sigma-Aldrich, St. Louis, MO). Soil Organic Carbon (SOC), organic matter, and elements N, H, and S were determined using a CHNS/O-2-Flash 2000 analyzer at the KAUST analytical core lab.

### DNA extraction and amplification for microbiome analysis

The gDNA of the compartments was extracted using the FastDNA Spin for soil, rhizosphere, and rhizoplane samples and Power Soil Pro and DNeasey Plant Pro Kit (MO BIO Laboratories, Carlsbad, CA, USA) for roots and leaves Samples, respectively. To homogenize bulk soil and endosphere samples, they were subjected to 1 cycle of bead beating for 2 min at 2000 rpm using the PowerLyzer24 Homogenizer (MO BIO Laboratories). The primer set of the amplification of V5-V7 was forward primer (799 F, 5′-AACMGGATTAGATACCCKG-3′) and reverse primer (1193R, 5′-ACGTCATCCCCACCTTCC-3′). Concentrations of extracted DNAs were quantified using the NanoDrop 2000 spectrophotometer (Thermo Fisher Scientific, Germany) and Qubit dsDNA high-sensitivity (HS) Kit (Thermo-Fischer Scientific). The quality control was performed by electrophoresis on 1% agarose gels and gDNA were stored at -20 °C until used.

### The cultural-dependent analysis: isolation of bacteria from mangrove

A serial dilution technique was employed to isolate bacterial communities from the bulk soil and three root compartments. The serial dilutions were then plated onto five different media for optimal bacterial isolation. The media utilized for bacterial isolation and purification were as follows: Seawater Luria-Bertani (SLB), Seawater yeast (SY) extract (L7025, Sigma-Aldrich), Tryptic Soy Agar (TSA and Zobell Marine (ZM) Agar. The last two media were prepared using filtered autoclaved seawater [25, 26]. The spread plates were incubated at 28 °C, and distinct colonies were selected over 7 days. These colonies were then sub-cultured twice for purification before being prepared as glycerol 25% stocks and stored at -80 °C. The appearance of each isolate’s colony morphology was recorded.

### Identification and taxonomic assignment of culturable bacteria

The cultural-dependent identification was conducted by extracting the total genomic DNA from the pure bacterial cultures growing on ZM Agar and incubated for 48 h at 30 °C. DNA extraction was performed using the Gen Elute™ Bacterial Genomic DNA Kit (Sigma Aldrich, Germany) according to the manufacturer’s instructions. The 16 S rRNA gene amplification was performed in thermal cycles using the universal primers set (F27,5′-AGAGTTTGATCMTGGCTCAG-3′) and (R1492, 5′-F27TACGGYTACCTTGTTACGACTT-3′) generating a fragment size ≈ 1500 bp. Following the standard protocol, the PCR product was purified with ExoSAP-IT™ Cleanup Reagent (Applied Biosystems) and then sequenced with both primer pairs using the Sanger technology 16 S rRNA sequencing at the Bioscience Core Lab (KAUST). The combined sequence was compared with related sequences from the database of GenBank (http://blast.ncbi.nlm.9nih.god//v/Blast.cgi). Based on the alignment of the complete 16 S rRNA gene sequence of the isolates, a phylogenetic tree showing the relationship among the strains was then constructed using the neighbour-Joining method /UPGMA method version 3.697.

### Assessment for plant growth promoting traits and tolerance to abiotic stresses

To determine the biochemical capabilities and plant growth promotion traits of the isolated bacterial collection, all the isolates were plated on Sea water LB agar and incubated at 30 ºC for 48 h and teste in triplicates. The siderophore production using Chrome Azurol Sulphonate (CAS), IAA production and phosphate solublization were measured according to [27], The proteolytic activity was evaluated by point inoculation of fresh bacterial culture on skimmed milk agar, and the plates were incubated at 30 °C for 48 h. After incubation, a clear hydrolytic zone of skimmed milk hydrolysis around bacterial isolates was considered positive, and no zone was considered negative [28]. To assess the ability of bacterial isolates to produce pectinases, fresh bacterial cultures were inoculated onto Czapek’s agar plates containing pectin as the sole carbon source (Czapek’s agar plates- Sigma) [29]. Bacterial cultures that developed a clear zone around the margins of their colonies were considered to have pectinase activity. Tolerance to salt and heat stress assays were performed by inoculating bacteria into 100 ml flask containing LB broth (Lennox L Broth Base, Invitrogen) amended with different concentrations of NaCl; 0.3, 0.6, 2, 3, and 5 M. At the same time, it was incubated at two different temperatures (37, and 45 ± 2 °C) on a rotary shaker (180 rpm) for 48 h at four different incubation temperatures. Bacterial growth was determined at OD600 to determine NaCl tolerance using Varioskan Flash.

**Plant assays using*Arabidopsis thaliana***.

A two-step screening process was conducted to evaluate the effects of isolated bacteria on *A. thaliana* Col-0 seedling growth under normal and salt stress conditions. Initially, 77 bacterial strains were assessed for plant growth promotion. Seeds were surface sterilized, rinsed, and dried. Bacterial strains were cultured in SLB medium, incubated, refreshed, and diluted to 10E9 CFU ml^− 1^ (OD 0.2 at 600 nm) before inoculation.

For the inoculation procedure, a 100 µl (10E9 CFU/ml) of the bacterial culture was mixed thoroughly with cooled 50 ml of half-strength Murashige and Skoog basal medium (MS) (Sigma-Aldrich, St. Louis, MO, USA) as described in [27]. Two methods were used: the Square Plate Method (SPM) with ½ MS and ½ MS with 100mM NaCl for stress conditions, and the Submerged Disk Method (SDM) using 24-well plates with agar discs amended with salt for stress conditions. Controls (MOCK) grew on ½ MS Agar with 100 µl SLB liquid. The fresh weight of plants was measured 14 days after transfer (DAT) and compared to MOCK (control) plates.

### Statistical and bioinformatic analysis

Microsoft Excel 2013 and GraphPad Prism 9 were used for statistical analysis. All the experiments were carried out in triplicate. The QIIME2 pipeline and R packages (v3.2.0) [30, 31] were used for microbiome analysis. The raw data of genomic microbiome DNA was merged and filtered to obtain clean reads. The DADA2 method was dereplicated or equivalent to 100% similarity clustering. Each de-duplicated sequence generated after noise reduction is called ASVs (Amplicon Sequence Variants). The obtained (ASVs) were annotated to obtain the corresponding species information and the abundance distribution based on the species. Alpha diversity analysis [32], including Chao1 for species richness, Simpson, goods coverage, and observed ASV’s, were calculated. A Venn diagram was generated to determine the common and unique ASVs information among different groups. On the other hand, multiple sequence alignments of ASVs perform and establish phylogenetic trees. Beta diversity analysis [33] was performed to evaluate the change in bacterial community structure based on principal coordinate analysis (PCoA) [34] that allow us to capture and represent the variation in microbial communities. In order to assign and visualize patterns in microbial community composition, we used Non-metric Multi-Dimensional Scaling (NMDS). NMDS is particularly effective for the analysis of microbiome data because it emphasizes rank-based dissimilarities between samples, allowing us to identify meaningful ecological gradients [35]. The differences among the groups in the microbial community structure are explained via dimension reduction analysis (PCoA and NMDS). The significant differentiation of bacteria structure among groups was assessed by ANOSIM [36] using QIIME2. The distance of the matrix was tested by ADONIS [37]. To further explore the differences in the community structure of different groups, statistical analysis methods such as T-test, MetaStat and LEfSe, were used to test the significance of differences in species composition and community structure of groups. The threshold on the Linear Discriminant Analysis (LDA) Effect Size (LDA score) [38] was set to equal or higher than 4.0. The annotation results of the amplicons were also associated with the corresponding functional databases. The PICRUSt2 software [39] was used to predict and analyse the function of the microbial community. The database of Clusters of Orthologous Groups of proteins (COGs) annotation scheme [40] was also used to capture the full breadth of functions within the microbiome of *A. marina*. Microbial co-occurrence networks for the ASVs mapping with cultivable strains were carried out with Spearman rank correlations (*r* > 0.8, or *r* < − 0.8; p < = 0.05) among microbial taxa. The nodes and edges in the constructed network represent taxa at the genus level. The modularity of the network is highlighted in different colours based on its complexity. Co-occurrence networks were visualized and property measurements were calculated using the Fruchtermann-Feingold layout of the interactive platform Gephi V 0.9.0 [41]. The bioinformatics analysis workflow after obtaining the sequencing data is shown in Figure S4.

### Data depositions

The 16 S rRNA gene sequences of the bacterial isolates in this study have been deposited in the GenBank database and are accessible under accession numbers (OR447762 - OR448017). 16 S microbiome sequence data generated in this study are available at NCBI under the BioProject ID PRJNA1063583.

## Results

### Sample collection and soil chemical properties

To better understand the chemical properties of mangrove soils and how they influence the mangrove ecosystem, we conducted a detailed analysis of soil samples from a mangrove site on the Red Sea shore. Our study aimed to answer which specific soil chemical characteristics exist in this mangrove environment, and how they vary between different compartments of the mangrove ecosystem. The mangrove-sampling site at KAUST on the Red Sea shore covers an area of 6,946.69 m², with an average soil temperature ranging from 25 to 35℃ in March 2019. The soil’s salinity was measured at 35 practical salinity units (PSU), and its electrical conductivity (EC) was 39 µS/cm at a pH of 7.5. The soil was characterized by a high content of macronutrients, including Calcium (Ca) and Magnesium (Mg); micronutrients, such as Iron (Fe); trace elements like Aluminium (Al) and Sodium (Na); and heavy metals, including Titanium (Ti) (Table S1). Our analysis revealed that the total carbon content was higher in the plant compartments (roots and leaves) compared to the soil, rhizosphere, and rhizoplane, with values of 32.2% and 29.26% in the roots and leaves, respectively, versus 8.04%, 3.07%, and 2.63% in the soil, rhizosphere, and rhizoplane. Nitrogen content was low in the bulk soil at 0.17%, while it was significantly higher in the leaves and roots, at 0.69% and 1.47%, respectively. Sulphur content was slightly elevated in the roots and leaves compared to the bulk soil, rhizosphere, and rhizoplane, with values of 2.64% and 2.35% versus 1.6%, 1.78%, and 1.84%.

Dissolved oxygen levels were adequate in the roots and leaves but lower in the bulk soil and reaching minimal values in the rhizosphere and rhizoplane. This suggests more anaerobic conditions in the latter compartments, with oxygen levels at 19.9% and 20.1% in the roots and leaves, compared to 12.1%, 3.96%, and 3.29% in the bulk soil, rhizosphere, and rhizoplane, respectively. In conclusion, our study highlights significant variability in the chemical properties of different compartments within the mangrove ecosystem (Table S1).

### Taxonomical Composition of *A. marina* Microbiome

To understand the taxonomical composition of the *A. marina* microbiome, we performed metabarcoding sequencing and analysis of bacterial communities associated with mangrove species. Our goal was to determine the diversity and abundance of bacterial taxa present in *A. marina* and to provide a comprehensive overview of its microbiome. PCR amplification targeting the V5-V7 regions of the 16 S rRNA gene resulted in two bands: one corresponding to bacterial 16 S rRNA and the other to 18 S chloroplast rRNA. The 500 bp band was excised from the gel and purified for further analysis (Figures S5, S6). Using the NovaSeq PE250 sequencing platform, we generated 3,972,677 reads with an average length of 377 bp. After applying quality filters and removing chimeras and non-target DNA, we obtained 4,012,921 effective sequences for subsequent analysis and annotation (Table S2).

We successfully annotated 19,678 ASVs, with Good’s coverage estimation and Q20 values above 98.0%, indicating sufficient sequencing depth for analysing bacterial communities. Based on taxonomic annotation, we identified the top 10 taxa at each taxonomic rank to create a distribution histogram of the relative abundance of taxa. Among the 25 samples, all ASVs were mapped to over 40 phyla. The most enriched phyla (relative abundance > 0.01%) included Proteobacteria, Firmicutes, Desulfobacterota, Bacteroidota, Actinobacteriota, Myxococcota, and Gemmatimonadota (Fig. 1C and Figure S7-A).

In all samples, bacterial communities were dominated by Proteobacteria, with the highest abundance found in roots (67%) and leaves (54%), followed by rhizoplane (50%), bulk soil (44%), and rhizosphere (36%). Firmicutes were most abundant in leaves, while Desulfobacterota and Bacteroidota were most prominent in the rhizosphere (13%). The rhizoplane had similar levels of Firmicutes, Desulfobacterota, and Bacteroidota (11%, 9%, and 9%, respectively). In roots, Firmicutes and Bacteroidota were also significant (8% and 7%, respectively). At the class level, bacterial communities across the five compartments were primarily composed of Gammaproteobacteria and Alphaproteobacteria. In bulk soil, these classes accounted for 27.7% and 17.4%, respectively; in the rhizosphere, 23.5% and 15.3%; in the rhizoplane, 31.7% and 20.3%; in roots, 47% and 21.3%; and in leaves, 43.9% and 11.3%. Bacilli were notably present in root and rhizosphere samples (17.9% and 12.3%, respectively), while Rhodothermia accounted for 6.7% and 6.5% in bulk soil and rhizosphere. Bacteroidia were found in the rhizosphere, rhizoplane, roots, and leaves samples (4.5–6.5%). Fewer bacterial communities were associated with classes like Desulfuromonadia, Actinobacteria, and Desulfobulbia, ranging between 1.5 and 2.3%.

At the order level, Oceanospirillales and Bacillales were dominant in all compartments except for the roots, where only Oceanospirillales prevailed (15.6%). In bulk soil, other notable orders included Rhodobacterales (4.1%), Rhodothermales (3.2%), Rhizobiales (2.6%), and Burkholderiales (2.5%). In the rhizosphere, Desulfobulbales (5.6%), Balneolales (4.7%), and Desulfobacterales (4.5%) were prominent. Rhizobiales (7.2%) and Burkholderiales (6.8%) dominated in the rhizoplane. In roots, Burkholderiales (11.4%), Rhizobiales (6.9%), and Pseudomonadales (6.9%) were significant, while leaves were dominated by Salinisphaerales (13.69%) and Burkholderiales (8.3%).

At the family level, Halomonadaceae were prevalent except in the rhizosphere and leaves, where they accounted for 8.6% in bulk soil, 9.6% in rhizoplane, and 13.6% in roots. Bacillaceae were dominant in the rhizosphere (11.3%) and bulk soil (8%). In leaves, Salinisphaeraceae (13.7%) and Bacillaceae (8.7%) were prominent. Oxalobacteraceae accounted for 6.4% in roots. High-resolution, culture-independent data revealed the relative abundance of specific genera. Salinisphaera in leaves (14%), Bacillus in the rhizosphere (9.6%), and Halomonas in bulk soil (7.5%) were the most prominent. Larsenimonas was notable in both roots and rhizoplane (9% and 7%, respectively). The least abundant genera included Streptococcus, Candidatus thiodiazotropha, and Corynebacterium in various compartments. Mid-range abundance genera included Brevundimonas in bulk soil (2.9%), Larsinemonas in the rhizosphere (2.9%), and Ralstonia in the rhizoplane (4%). In conclusion, our taxonomic analysis of the *A. marina* microbiome highlights the diversity and distribution of bacterial communities across different mangrove compartments.

### The diversity of bacterial taxa

**Fig. 2:**
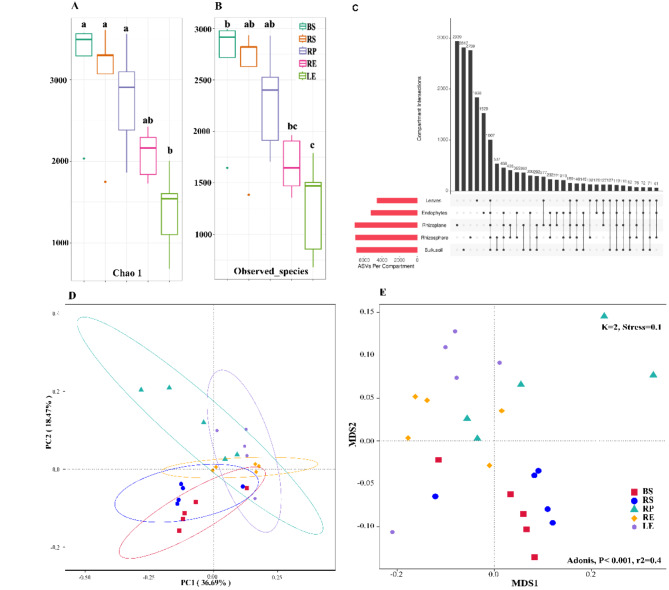
Microbial diversity and community structure across different *Avicennia marina* compartments. (**A**) Alpha diversity comparison using the Chao1 index, indicating species richness across bulk soil (BS), rhizosphere (RS), rhizoplane (RP), root endosphere (RE), and leaf endosphere (LE) compartments. Letters above boxplots denote statistically significant differences (*p* < 0.05). (**B**) Alpha diversity comparison using the Observed Species metric, highlighting variations in species richness among compartments, with significant differences denoted by letters. (**C**) UpSet plot illustrating the unique and shared amplicon sequence variants (ASVs) among different compartments, emphasizing compartment-specific microbial communities. (**D**) Principal Coordinate Analysis (PCoA) plot showing the clustering of microbial communities based on Bray-Curtis dissimilarity, with ellipses representing 95% confidence intervals. (**E**) Non-metric Multi-Dimensional Scaling (NMDS) analysis depicting the distribution of microbial communities, with an Adonis test result (*p* < 0.001, r² = 0.4) confirming significant differences among compartments

To distinguish between common and unique bacterial taxa across different compartments (Bulk soil, Rhizosphere, Rhizoplane, Roots, and Leaves), an UpSet plot was used. Bars represent the number of ASVs, highlighting how certain ASVs are exclusive to specific compartments (e.g., leaf endosphere) while others are shared among multiple compartments, illustrating the degree of microbial overlap. Our analysis revealed that the five compartments shared 1,007 (5.1%) of a total of 19,749 ASVs. Specific ASVs were identified for each compartment: 2,759 (13.9%) in the Rhizosphere, 2,939 (14.9%) in the Rhizoplane, 1,529 (7.7%) in the Roots and 1,838 (9.3%) in the Leaves (Fig. 2C). At the genus level, some of the common ASVs included Acinetobacter, Bacillus, Brevundimonas, Candidatus, Halomonas, Pontibacillus, Streptococcus, Massilia, and Salinisphaera. Interestingly, the higher number of ASVs in the bulk soil and rhizosphere reflects the diverse and heterogeneous microbial populations present in these compartments. These environments are likely subject to various external influences, such as soil properties, nutrient availability, and environmental conditions. The presence of unique ASVs in the root and leaf endospheres indicates that these compartments harbor specialized microbial communities. This specialization may be driven by the plant’s immune system, nutrient availability, and specific biochemical signals released by *A. marina*, which select for microbes that can aid in stress tolerance or nutrient uptake. The significant overlap of ASVs between the rhizosphere and rhizoplane highlights the continuity of microbial communities influenced by root exudates and physical proximity to the root surface. This overlap suggests a gradient of microbial interactions transitioning from the bulk soil to the root surface.

To assess the bacterial diversity in each compartment, we used several alpha diversity indices to estimate Chao1 (diversity) and Observed ASVs (richness) (Fig. 2A and B). The diversity of bacterial species decreased from bulk soil (3,000 ASVs) to the Rhizoplane and both Roots and Leaves (~ 1,500 ASVs). The observed species richness was lowest in the Rhizoplane and endophytes (Roots and Leaves) compared to the bulk soil, indicating a lower number of unique bacterial species in these plant-associated compartments. This suggests a reduced diversity and potentially a more specialized microbial community in these areas.

Good’s coverage values were above 0.95, indicating that the sequencing effort covered a substantial proportion of the actual bacterial diversity present in the samples, providing a reliable estimate of species richness. Both Roots and Leaves had significantly lower richness (Chao1 value) than the other groups (*p* < 0.05) (Table S3).

Beta diversity was analysed to assess further the differences between the bacterial communities by PCoA and NMDS. The scatter plot, depending on the PCoA scores, showed that the composition of the population differs among the groups, with some overlaps between Rhizoplane and all other compartments (Fig. 2D). The two principal coordinates were responsible for the variance of 36.69% and 18.47%, respectively. The samples from BS and RS were relatively concentrated in the area below the vertical axis’ middle line. In contrast, the samples from the other three groups were concentrated in the area above. Furthermore, the variations within the group were visualized by NMDS (Fig. 2E), in which the samples were separated according to origin (*P* < 0.05, Adonis test) as shown in the Table S4 and Figure S8. The NMDS plot includes the results of an **Adonis (PERMANOVA) test**, which statistically assesses the significance of differences in microbial community composition among compartments. The p-value (< 0.001) and r² value (0.4) indicate that the differences in community composition between compartments are highly significant, with compartment type explaining 40% of the variation in the data (Fig. 2E). Using the pairwise Bray-Curtis similarity index, the estimated distance between each two samples was small enough to aggregate into clusters except for rhizoplane, which was scattered among the other groups. The stress levels of the NMDS plot are below 0.2, indicating a good representation of the original data. This was substantiated by the more detailed analysis of similarity (*P* < 0.05, MRPP test in Table S5). The AMOVA analysis (Table S6) shows that the bacterial community structures of the compartments differ significantly from each other (*p* < 0.05) except for Bulk soil and Rhizosphere (*P* = 0.212), indicating that the differences amongst these two groups are very low. The NMDS plot shows a clear separation between microbial communities from different compartments, highlighting the significant compartmentalization of microbial diversity. For example, bulk soil (BS) and rhizosphere (RS) samples are more distinct from root (RE) and leaf endosphere (LE) samples, suggesting that environmental factors and plant influence the microbial community structure differently across these compartments. (Fig. 2E). Overall, the separation pattern indicated distinct microbial community composition between compartments.

### The compartment biomarker taxa

**Fig. 3:**
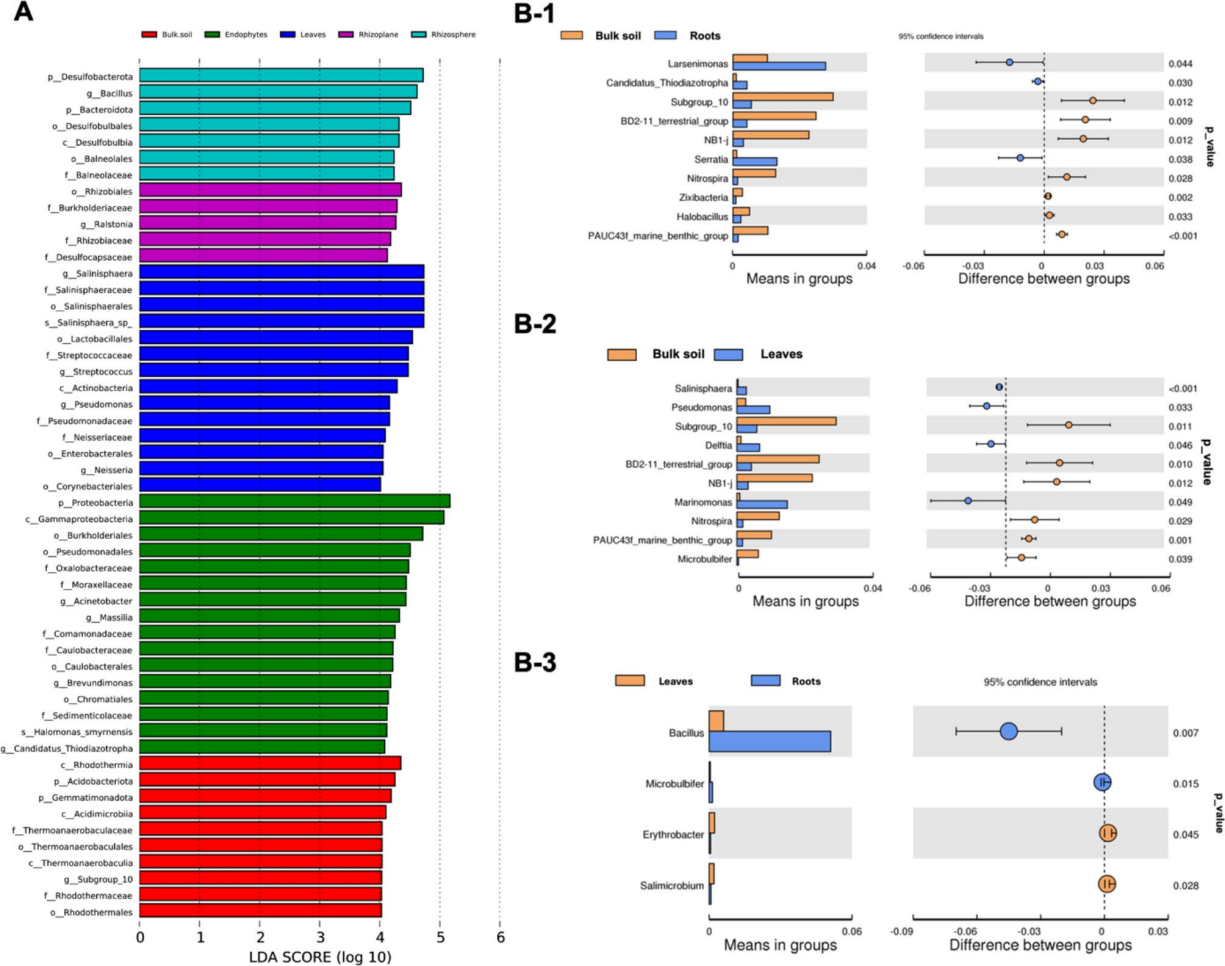
**A**) Histogram of linear discriminant analysis (LDA) score of LEfSe analysis higher than 4 of the bacterial communities between Roots (green), Leaves (blue), Rhizoplane (magenta), and Rhizosphere (cyan), and Bulk soil (red). The bar graph shows LDA scores for phytoplankton taxa. Only taxa meeting an LDA significant threshold > 2.0 are shown. The LDA score on the log_10_ scale is indicated at the bottom. The greater the LDA score is, the more significant the phylotype biomarker is in the comparison. **B**- Comparative taxonomic profiles at the genus level. (B-1) Bulk soil-roots, (B- 2) Bulk soil-leaves, and (B- 3) Roots-Leaves. The left panel is the abundance of species showing significant differences between groups. Each bar represents the mean value of the abundance in each species group, showing significant differences between groups. The right panel is the confidential interval of between-group variations. The left-most part of each circle stands for the lower 95% confidential interval limit, while the right-most part is the upper limit. The centre of the circle stands for the difference in the mean value. The circle’s colour agrees with the group whose mean value is higher. The right-most value is the p-value of the significance test of between-group variations

To identify each compartment biomarker taxa, LEfSe analysis was performed to compare the relative abundance (differential abundance between the groups with LDA score > 2 and *P* < 0.05). Our results emphasize that phylum; order, family, and genus were mainly potential biomarkers for different compartments. The relative abundance counts of *Rhodothermia*, *Bacillus*, Burkholderiaceae, Proteobacteria, and *Salinisphaera* were over-represented. Six to sixteen unique bacterial taxa were found for each compartment. LEfSe analysis indicated that the orders Thermoanaerobaculaceae and Rhodothermales were higher in the Bulk soil (Fig. 3A), while the genus *Bacillus* and orders Desulfobulbales, and Balenolales were higher in Rhizosphere. In the Rhizoplane, the genus *Ralstonia*, order Rhizobiales, and family Burkholderiaceae were enriched (LDA scores > 4.0). The bacterial groups enriched in Root included species *Halomonas smyrnensis*, the genera *Acinetobacter*, *Massilia*, *Brevundimonas*, and Candidatis, Oxalobacteriaceae and Moraxellaceae at the family level, Burkholderiales, and Pseudomonadales at the order level, and Proteobacteria, Gammaproteobacteria, Alphaproteobacteria at the phylum level (LDA scores > 4.0). The genera *Salinisphaera* and *Streptococcus*, the orders Salinisphaerales and Lacatobacellales, and the families Salinisphaeraceae and Streptococcaceae were biomarkers for the Leaf community (Fig. 3A).

Genera with significant differences in abundance among groups and intra-group were captured, and their distribution among groups was assessed. A comparison of intra-group and inter-group variances was calculated to determine the significance of inter-group variances at the genus level using the t-test. Compared to Bulk soil, the roots were significantly enriched in *Larsenimonas* and *Serratia* (*P* < 0.05). At the same time, the leaves bacterial community shows a significant increase in the abundance of *Salinisphaera* (*P* < 0.001), *Pseudomonas* (*P* < 0.05), *Delftia* (*P* < 0.05), and *Marinomonas* (*P* < 0.05). Minor significant differences were found between the ASVs of the roots and leaves endophytic communities such as *Bacillus* and *Microbulbifer* in the roots while *Erythrobacter* and *Salimicrobium* in the leaf’s samples (*P* > 0.05) (Fig. 3B, C, and D). These findings highlight the unique microbial signatures of each compartment, indicating specialized microbial communities that adapt to the specific conditions of their respective niches. This compartment-specific microbial distribution enhances our understanding of the ecological interactions within mangrove ecosystems and provides potential biomarkers for environmental and ecological studies.

### The microbiome function prediction

**Fig. 4:**
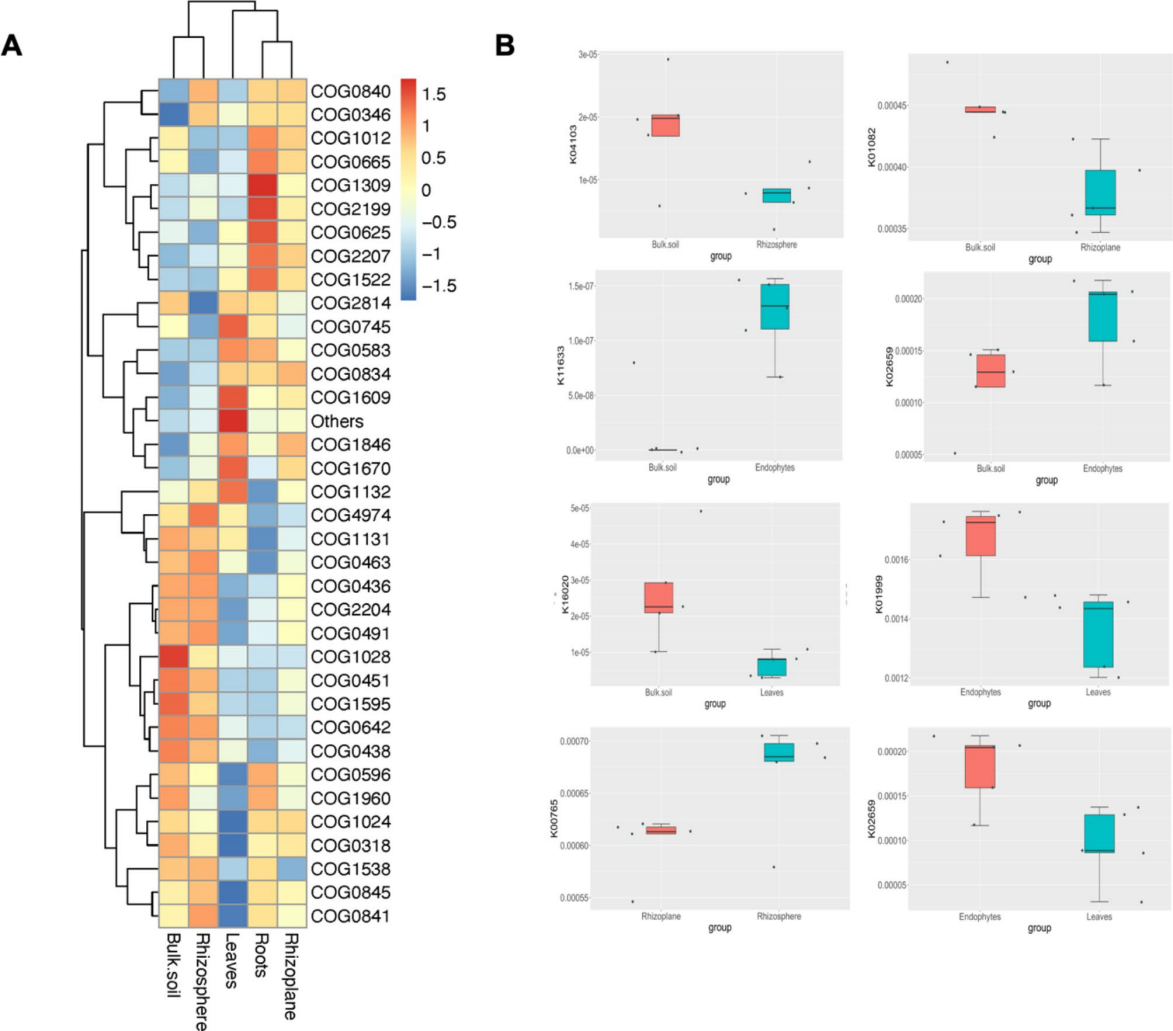
Functional gene profiles and differential abundance across*Avicennia marina* compartments: (**A**) The heatmap displays the relative abundance of Clusters of Orthologous Groups (COGs) in bulk soil, rhizosphere, rhizoplane, roots, and leaves. The color gradient ranges from blue (low abundance) to red (high abundance), highlighting how microbial communities adapt functionally to different microhabitats. Hierarchical clustering reveals compartment-specific patterns of microbial gene expression, emphasizing the specialized roles of these communities in the mangrove ecosystem. (**B**) The series of boxplots compares the abundance of selected KEGG Orthology (KO) genes between specific compartments. Each boxplot represents a different gene and shows how its abundance varies between compartments, such as bulk soil vs. rhizosphere, bulk soil vs. rhizoplane, or endophytes vs. leaves. The x-axis labels indicate the compartment groups, while the y-axis displays gene abundance. Significant differences in gene abundance are highlighted, suggesting distinct microbial functional roles that contribute to plant growth and stress resilience in varying environmental conditions

To understand the functional potential of bacterial communities in different compartments of *A. marina*, we conducted a detailed functional annotation analysis focusing on the top 10 Clusters of Orthologous Groups (COG) (Fig. 4A). This analysis aimed to predict the metabolic and functional capabilities of the microbiome associated with *A. marina*. In the bulk soil, NAD(P)-dependent dehydrogenase proteins (COG1028) were significantly enriched, indicating robust metabolic activity related to oxidation-reduction processes. In the rhizosphere, site-specific recombinase XerD (COG4974) was highly enriched, suggesting an active role in recombination processes essential for chromosome segregation during cell division. Additionally, histidine kinase BaeS (COG0642) was significantly represented, highlighting its role in signal transduction and environmental adaptation.

The rhizoplane exhibited slight representation of COG2207 (involved in sugar metabolism), COG0834 (related to amino acid transport and signal transduction), and COG1846 (a transcriptional regulator involved in detoxification of metalloids and degradation of organic compounds). In the roots, several COGs were notably prevalent. COG1012, involved in metal detoxification and organic biodegradation, plays a crucial role in converting benzyl-alcohol through benzaldehyde into benzoate. COG1309 (TetR/AcrR family transcriptional regulator) is involved in DNA-binding transcriptional regulation and fatty acid biosynthesis, while COG0583 (LysR family transcriptional regulator) is found in both roots and leaves, indicating its role in transcriptional regulation. COG0596 (alpha/beta hydrolase superfamily gene) is highly represented in bulk soil and roots, reflecting its role in hydrolysis and transfer reactions. COG0625 (glutathione S-transferase gene) is expressed in roots, involved in detoxification processes, and COG0745 (response regulators of a CheY-like receiver domain) is highly represented in leaves, playing a role in DNA to RNA transcription. In leaves, COG1670 (acetyltransferase GNAT9 domain) is involved in posttranslational modification and protein turnover, while COG1609 (catalysis of DNA to RNA transcription proteins) is responsible for sugar metabolism, similar to COG2207 in the rhizoplane and roots.

The KEGG pathway analysis revealed significant differences across compartments, indicating compartment-specific metabolic capabilities. In bulk soil, pathways related to tryptophan metabolism (K04103), proteasome (K13527), and carbon fixation (K15232) were significantly enriched, suggesting active tryptophan biosynthesis and degradation, protein turnover, and carbon assimilation, respectively. In the rhizosphere, nitrate reduction (K07696) and sulfur metabolism (K01082) pathways were highly enriched, emphasizing roles in nitrogen cycling and sulfur assimilation. In the rhizoplane, pathways for amino sugar and nucleotide sugar metabolism (K18675) and nitrogen metabolism (K15371) were enriched, highlighting roles in cell wall synthesis, modification, and nitrogen transformation.

Roots showed significant enrichment in pathways related to biofilm formation (K02659), indicating microbial community stability and protection, and multidrug resistance (K18901), highlighting adaptation to stress and antibiotic resistance. ABC transporters (FrcC, K10553) were enriched, reflecting active nutrient uptake and transport. Leaves had significant enrichment in pathways related to amino sugar and nucleotide sugar metabolism, and ABC transporters (BcrA; bacitracin transport system ATP-binding protein, K19309), indicating active transport and defense mechanisms. Butanoate metabolism (K18382) was also enriched, reflecting metabolic versatility and energy production. These data reveal a specialized metabolic and functional role of bacterial communities in different compartments of the *A. marina* ecosystem. The differential abundance of COGs emphasizes the adaptation of microbial communities to specific microhabitats within the mangrove ecosystem. For instance, microbes in the rhizosphere are likely equipped with genes for efficient nutrient uptake and stress response, while those in the endosphere may be more specialized for symbiotic interactions and promoting plant health. and resilience of mangrove ecosystems.

### Mangrove bacterial cultural, characteristic, PGP traits and stress tolerance

**Fig. 5:**
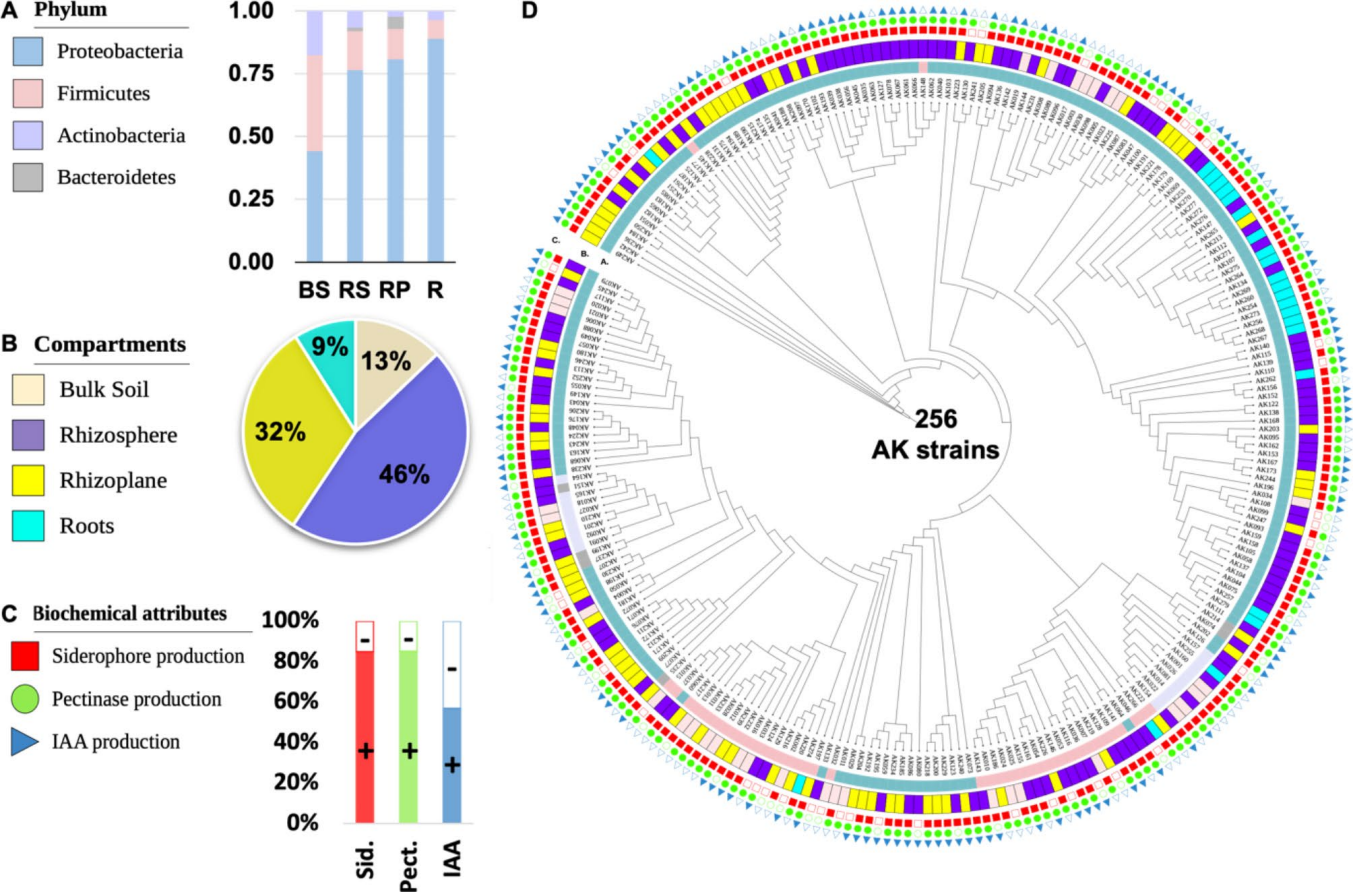
Cultured bacteria collection and functional attributes across *Avicennia marina* compartments (**A**) The bar plot shows the distribution of the 256 cultured bacterial isolates across different phyla, including Proteobacteria, Firmicutes, Actinobacteria, and Bacteroidetes, providing an overview of the taxonomic diversity of the isolates. (**B**) The pie chart illustrates the distribution of the 256 isolates among the various compartments of origin, including bulk soil (9%), rhizosphere (13%), rhizoplane (46%), and roots (32%). This emphasizes the relative abundance of cultured isolates across different *Avicennia marina* microhabitats. **C**)This panel highlights the common plant growth-promoting (PGP) traits exhibited by the isolates, such as: Siderophore Production (red): Facilitating iron acquisition for plants. Pectinase Production (green): Enabling the breakdown of plant cell wall components. IAA Production (blue): Promoting plant growth through phytohormone synthesis. The “+” and “-” symbols indicate the presence or absence of these PGP traits among the isolates **D**) The circular phylogenetic tree depicts the relationships among the 256 cultured bacteria based on 16 S rRNA gene sequencing. The tree provides a comprehensive view of the phylogenetic diversity of the mangrove-associated bacteria, with branches color-coded according to the phyla and compartments of origin, linking taxonomic identity to ecological niche

To capture the mangroves cultivated bacteria (AK) a culture dependent method was used to isolate a total of 256 bacterial strains from different compartments of A. *marina* plants and bulk soil. These isolates were distributed as following: Bulk soil (33 isolates, 13%), Rhizosphere (119 isolates, 46%), Rhizoplane (81 isolates, 32%), and Roots (23 isolates, 9%) (Fig. 5B and Figure S11-A). In order to characterize the isolated bacterial strains, several morphological and biochemical test were conducting including colony morphology, shape, margin, color, transparency, texture, and size of the colonies grown on SLB agar. The isolated collection of bacterial strains showed varying preferences for different types of agar media. The Zobell Marine (ZM) Agar was the most favourable, with 29% of the isolates growing on this media. This was followed by R2A + 0.5 M NaCl Agar (23%), Seawater Luria Bertani (SLB) Agar (21%), and Seawater Yeast (SY) Agar (18%). TSA was found to be the least favourable medium, as only 9% of the isolates grew on it (Figure S11-B).

Biochemical characterizations were conducted on the isolated bacteria to detriment the activities, the isolated bacteria were found to possess several plant growth-promoting (PGP) traits, which can significantly affect plant growth directly and indirectly (Fig. 5C and Figure S13, Table S7). Notably, a high percentage of the bacterial mangroves’ isolates exhibited siderophore production (84.8%) and pectinase hydrolysis (85.2%) ability. These abilities are crucial for nutrient uptake and degradation of complex compounds in the plant environment. Another noteworthy finding was that approximately 57% of the isolates could produce indole-3-acetic acid (IAA). IAA is a well-known plant hormone that is vital in promoting root growth and overall plant development. Furthermore, although the proteolytic and cellulose activity of the isolates was relatively lower (38.73% and 22.3%, respectively).

In terms of stress tolerance, the AK isolates demonstrated remarkable capabilities as the majority (93%) were found to be halotolerant, capable of thriving in the presence of high concentrations of NaCl in the growth medium. Remarkably, 50% of the isolates were identified as extreme halophiles, displaying excellent growth ability even in environments with 29% and 17.5% (5 M and 3 M) NaCl, respectively. Additionally, a significant percentage (89.1%) of the isolates exhibited robust growth even in the presence of 11% (2 M) NaCl, indicating their remarkable salt tolerance. Moreover, the isolates showed a strong capacity for thermophilic growth. A vast majority (92.2%) thrived well at 37 °C, while a significant proportion (66.8%) also exhibited growth at a higher temperature of 45 °C. These findings highlight the adaptability and resilience of the AK isolates to different temperature regimes (Fig. 5C, Figure S13 and Dataset).

### Phylogenetic analysis and data deposition

The pure bacterial isolates were further characterized through phylogenetic analysis using 16 S rRNA gene sequences (Fig. 5D). The AK bacterial isolates were found to span 34 genera within four phyla: Proteobacteria (α and γ) accounted for 73.8% of the isolates, followed by Firmicutes (Bacilli) (17.2%), Actinobacteriota (6.6%) and Bacteroidota (Cytophagia and Flavobacteria) 2.3% (Fig. 5A and Supp. Figure S14). The main bacterial community for each compartment was also annotated at the genus level depending on the full-length 16 S rRNA sequences (Supp. Figure 12 A-D).

### The network association analysis of the recovered bacterial genera

**Fig. 6:**
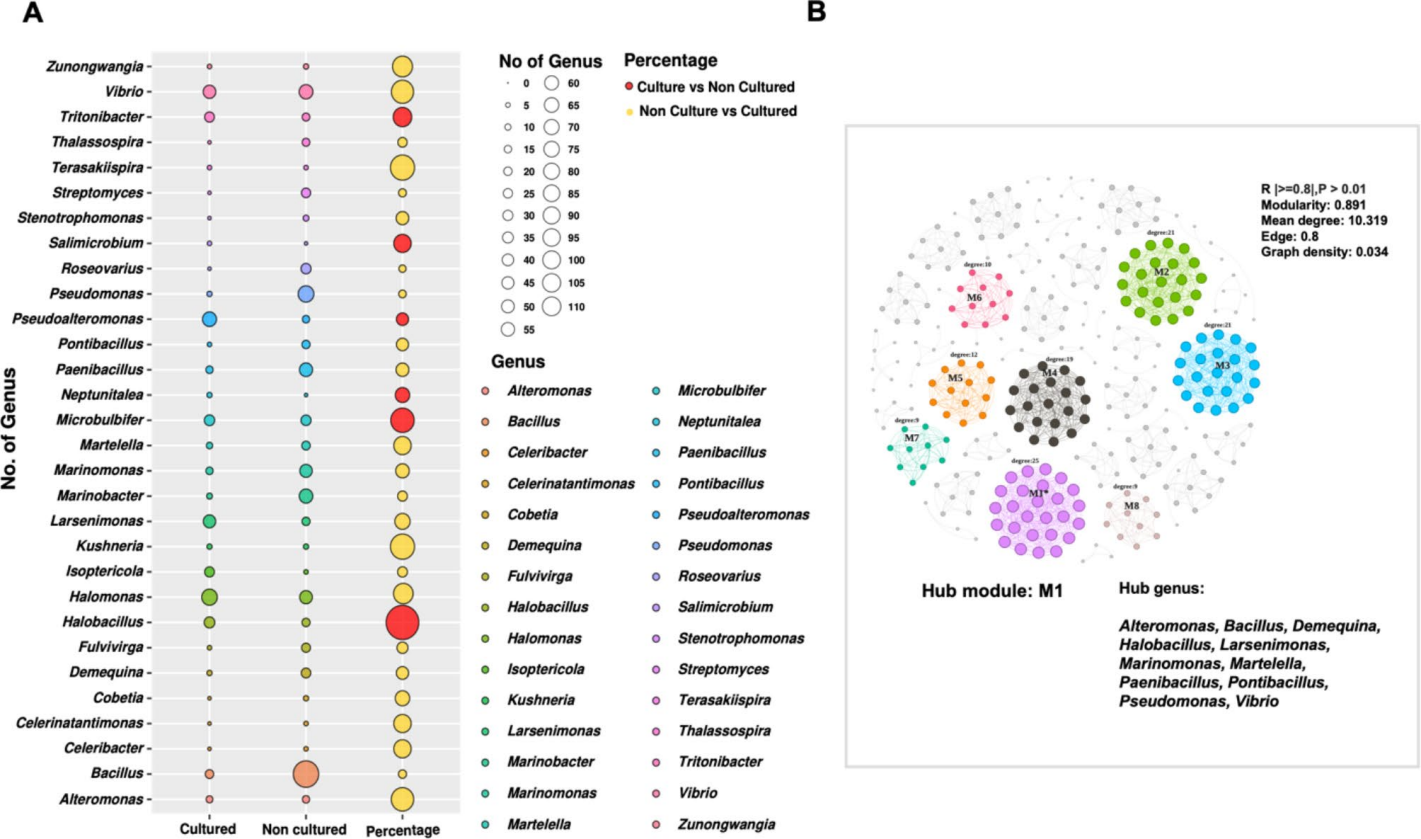
Genus-level distribution and network analysis of cultured bacteria from *Avicennia marina* (**A**) **The bubble plot: the bubble plot** represents the distribution of bacterial genera across different *Avicennia marina* compartments, with each row corresponding to a specific genus. The size of the bubbles indicates the relative abundance of each genus, with larger bubbles representing higher abundance. The x-axis denotes the different compartments, while the y-axis lists the bacterial genera. The color of each bubble corresponds to the genus, as indicated by the color legend on the right, providing a visual summary of how bacterial communities are distributed throughout the mangrove microhabitats. bubble size guide, indicating the relative abundance values associated with each bubble size, ranging from 0 to 110. (**B**) **Network Analysis** The network diagram illustrates the co-occurrence relationships among bacterial genera isolated from *Avicennia marina*. Nodes represent bacterial genera, and edges (connecting lines) indicate significant co-occurrence interactions (*p* < 0.01). The nodes are color-coded to match the genera in the bubble plot, and the size of each node reflects the abundance of the genus. The network layout highlights clusters of closely associated bacteria, suggesting potential functional or ecological interactions within the mangrove microbiome

The relative abundance of the genera among compartments showed that the distribution of genera varied between the culture-dependent and culture-independent analyses (microbiome). However, some of the top 10 abundant genera in the culture-independent were also captured in the culture-dependent method, such as *Larsenimonas*, *Bacillus*, and *Halomonas* (Fig. 6A and Figure S15). Concerning the main selective criteria in our cultural-dependent methods, the genera recovered in the cultural-dependent analysis were either halophilic or halotolerant as they could grow at high NaCl concentrations (1–5 M NaCl) and the high temperature reaching 45℃. For example, *Microbulbifer* spp. was most abundant in bulk soil samples (35%); whereas *Halomonas* spp. dominated, rhizosphere samples (21%). *Bacillus* spp. was dominant in the rhizoplane (23%). In the Roots, *Larsenimonas* sp. was the most abundant genera (67%).

Hierarchical clustering of the taxa further indicated the importance of using selective media containing NaCl or seawater salts. Although some bacteria, such as *Bacillus* sp., can grow on several media, others, such as Actinobacteriota (*Isoptericola* sp., *Demequina* sp., and *Streptomyces* sp.), showed selective growth on media containing either high NaCl concentrations or seawater salts. While *Fulvivirga* sp. (Bacteroidota; Cytophagia) was recovered from R2A + 0.5 M NaCl and Zobell marine agar; *Neptunitalea* sp. (Bacteroidota; Flavobacteriaceae) was recovered from TSA and R2A + 0.5 M NaCl agar; conversely, *Salimicrobium* (Firmicutes) and *Cobetia* (Gammaproteobacteria) were recovered exclusively from SWY and SWLB agar, respectively.

To determine the keystone taxa among the cultivated strains, the association network analyses of the same genera recovered were used to highlight the microbial hubs in Module 1 (Fig. 6B) consisting of the genera *Alteromonas*,* Bacillus*,* Demequina*,* Halobacillus*,* Larsenimonas*,* Marinomonas*,* Martelella*,* Paenibacillus*,* Pontibacillus*,* Pseudomonas*, and *Vibrio.*

### Promotion of salinity stress tolerance on Arabidopsis

**Fig. 7:**
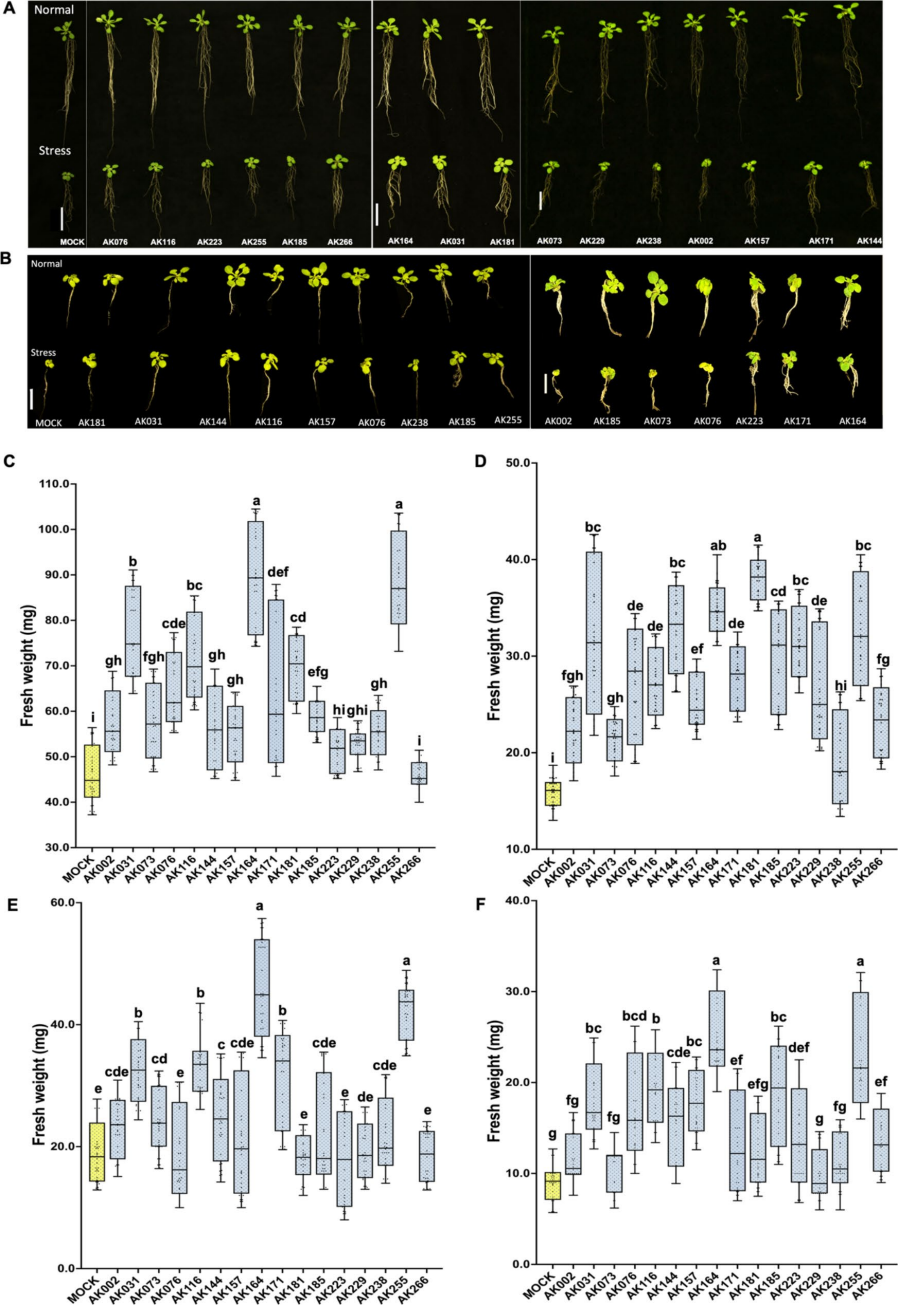
Growth Enhancement of *Arabidopsis thaliana* Seedlings by Candidate Bacteria Seedling Growth on Square Plate Method (SPM) and Submerged Disk Method (SDM) Plates: **A**- Images show *A. thaliana* seedlings inoculated with candidate bacteria versus non-inoculated controls (MOCK) under both normal (½ MS) and Saline Stress conditions (½ MS + 100mM NaCl) using the **SPM methods**. **B**- Images show *A. thaliana* seedlings inoculated with candidate bacteria versus non-inoculated controls (MOCK) under both normal (½ MS) and Saline Stress conditions (½ MS + 100mM NaCl) using the **SDM methods.** Bacterial inoculation visibly enhances root and shoot growth, especially under saline stress. **C**-**F**) **Box Plots**: plots of the fresh weight mean of *A. thaliana* grown NaCl after inoculated with the candidate bacteria in comparison with non-inoculated (MOCK) plants. C- SPM on 1/2MS, D- SPM on 1/2MS + 100mM. E- SDM on 1/2MS, F- SDM on 1/2MS + 100mM. Inoculated seedlings generally show higher fresh weight than MOCK, demonstrating the growth-promoting effects of candidate bacteria, particularly under saline stress

Seventy-seven isolates were chosen based on their source compartment, taxonomic diversity, and genera network correlation data, highlighting pivotal taxa in the ASVs network analysis of the microbiome (Fig. 6B). An initial screening relied on assessing fresh weight (FW) after a 20-day growth period were done and among these isolates, forty-two showed promising potential by significantly enhancing of *A. thaliana* growth under salt stress conditions (½MS with 100mM NaCl), demonstrating a beneficial increase of over 20% compared to the control (noted in Table S7). Subsequently, a second screening focused solely on strains that consistently exhibited plant growth-promoting effects across three independent experiments. Sixteen strains out of the initially selected 42 isolates (highlighted in Table S7) consistently demonstrated beneficial effects on plant growth compared to controls, both under normal and salt stress conditions. Employing the Square Plate Method (SPM) plant assay, nine of these sixteen mangrove strains notably increased the fresh weight of *A. thaliana* significantly, even under normal and stress conditions (Underlined in Table 1). However, seedlings were subjected to the Submerged Disk Method (SDM) plant assay for a more accurate simulation of the hypoxic and salt stress conditions found in natural mangrove ecosystems. Out of the 16 tested strains ( Figure S16-S19 ), five AK strains (AK031, AK116, AK164 [42], AK171, AK225) exhibited significant enhancement (> 20%) in plant growth compared to non-inoculated (MOCK) plants (highlighted in Table 1) by using all the assessments of SPM and SDM on normal and stress conditions, as depicted in Fig. 7.

**Table 1:** The beneficial increase (%) of *A. thalian*a’s fresh weight (gm) in the second screening for 16 candidates using SPM and SDM, *Paenibacillus lautus* (AK002 - OR447938), *Bacillus seohaeanensis* (AK031 - OR447774), Thalassospira tepidiphila MCCC 1A03514 (AK073 - OR447979), *Tritonibacter mobilis*(AK076 - OR447785), *Halobacillus locisalis* (AK116 - OR447813), *Microbulbifer elongatus* (AK144 - OR447909), *Demequina activiva* (AK157 - OR447779), *Isoptericola sp.* (AK164 - OR447867), *T. mobilis* (AK171 - OR447787), *Pseudoalteromonas flavipulchra* (AK181 - OR447934), *T. mobilis* (AK185 - OR447793), *Pseudomonas azotoformans* (AK223 - OR447928),, *Martelella mangrovi* (AK229 - OR447916), *Celerinatantimonas diazotrophica* (AK238 - OR447776), *I. chiayiensis* (AK255 - OR447854), *P. lautus* (AK266 - OR448017**)**

No.	Strain	SPM				SDM	
		½ MS	½ MS + 100mM NaCl		½ MS		½ MS + 100mM NaCl
*1*	*AK002*	26.3*	42.2****	9.5			21.8
*2*	*AK031*	49.8****	134.4****	45.8****			96.1***
*3*	AK073	15.6	37.6****	12.8			24.4
*4*	*AK076*	23.0**	69.3****	-8.2			87.3****
*5*	*AK116*	45.6****	94.5****	43.0***			125.2***
*6*	AK144	15.5	105.9****	17.5*			59.7**
*7*	*AK157*	32.2*	62.5****	-1.9			78.5****
*8*	*AK164*	124.9****	110.4****	107.0****			165.3****
*9*	*AK171*	27.8*	33.6**	35.2*			47.3*
*10*	*AK181*	35.5****	109.1****	-20.7			57.4*
*11*	AK185	16.0	46.6***	1.6			102.5****
*12*	AK223	9.9	47.9****	-10.1			58.0*
*13*	AK229	22.5	52.6****	-3.1			11.2
*14*	AK238	8.2	2.6	6.9			17.9
*15*	*AK255*	135.0***	111.2****	101.4****			146.1****
*16*	AK266	10.9	43.4***	9.5			48.7**

*p > 0.05, **P > 0.01, *** *P* > 0.001, **** *P* > 0.0001

## Discussion

### Microbial diversity and mineral cotenants

Mangrove ecosystems thrive in extreme saline environments and are crucial for coastal protection. These unique ecosystems stabilize shorelines by trapping sediments with their complex root systems, reducing erosion, and mitigating the impact of storm surges. Mangroves also enhance water quality by filtering pollutants and provide critical habitat for a diverse array of marine and terrestrial species, contributing significantly to coastal biodiversity. Their ability to sequester carbon further underscores their importance in combating climate change [43–45]. The feature of mangrove resilient to an extreme environment highlighted by their unique microbiome communities of a combination of marine and terrestrial microbes. The diverse microbial communities associated with mangrove roots, soil, and sediments, play a pivotal role in maintaining the health and resilience of mangrove ecosystems [46]. To elucidate the ecological importance of the microbiome in the mangrove ecosystem, we investigated the microbial diversity and functional potential of the *A. marina* microbiome across different compartments on the Red Sea shore and correlate with physical and chemical properties of the soil. Elemental analysis revealed the presence of various metals in the bulk soil and plant compartments, reflecting both natural and anthropogenic influences. The accumulation of metals such as Ag, Cr, Ni, As, and V in roots and Ti, Al, Sr, Ba, and Li in leaves suggests that *A. marina* has mechanisms for metal uptake and translocation. This metal accumulation indicates potential phytoremediation capabilities, where mangroves could be used to remediate contaminated soils and water. However, the ecological implications of metal accumulation need careful consideration, as excessive metal levels could adversely affect plant health and ecosystem functions. The ability of mangroves to tolerate and accumulate metals highlights their ecological resilience and potential use in bioremediation strategies [47]. Notably, the Rhizosphere and Rhizoplane exhibit significantly lower sodium content compared to other compartments, suggesting potential mechanisms regulating sodium concentrations or unique microbial interactions. These compartments also display higher bacterial diversity (Figs. 1 and 5), indicating a diverse range of species with potential specialized functions that may contribute to plant health and stress tolerance. The symbiotic relationship between *A. marina* leaves and endophytic bacterial communities, particularly Proteobacteria (Fig. 1C), might play a role in managing absorbed salts, influencing salt stress in leaves. Additionally, increased oxygen content in roots and leaves implies distinctive aerobic conditions. While chemical analyses provide insights, further exploration into specific bacterial capabilities is crucial.

### Microbial diversity and its ecological implications

The microbial communities associated with *A. marina* in the Red Sea region demonstrate significant compartmentalization, with distinct microbial populations in the bulk soil, rhizosphere, rhizoplane, roots, and leaves. This compartmentalization suggests specialized roles and adaptations of microbial communities to different plant microenvironments. For example, bulk soil and rhizosphere reflects the diverse and heterogeneous microbial populations present in these compartments. These environments are likely subject to various external influences, such as soil properties, nutrient availability, and environmental conditions. On the other hand, specialized communities in the root and leaf endospheres were observed. The presence of unique ASVs in the root and leaf endospheres indicate that these compartments harbor specialized microbial communities. This specialization may be driven by the plant’s immune system, nutrient availability, and specific biochemical signals released by *A. marina*, which select for microbes that can aid in stress tolerance or nutrient uptake. In addition, a clear overlap and crosstalk between compartments is evident. For example, in the case of microbial overlap between rhizosphere and rhizoplane highlights the continuity of microbial communities influenced by root exudates and their physical proximity to the root surface. This overlap suggests a gradient of microbial interactions transiting from the bulk soil to the root surface.

Proteobacteria, the dominant phylum across all compartments, is known for its diverse metabolic capabilities and ecological functions, including nutrient cycling, plant growth promotion, and disease suppression [48, 49]. The high prevalence of Proteobacteria underscores their importance in supporting *A. marina* health and resilience. The rhizosphere and rhizoplane exhibited higher bacterial diversity compared to other compartments, indicating these zones as hotspots for microbial activity and interactions. The lower sodium content in these compartments suggests that specific microbial processes or plant-microbe interactions are involved in regulating sodium levels, potentially mitigating salt stress and enhancing plant tolerance to high salinity conditions. d This finding is consistent with previous research that emphasizes the crucial role of rhizosphere and rhizoplane microbial communities in enhancing plant tolerance to environmental stresses [5, 9].

The consistent presence of dominant taxa like *Larsenimonas* and *Bacillus* across compartments indicates their adaptability and potential importance within this ecosystem. These results support the hypothesis that focused root-associated metagenomic analyses effectively unveil compartment-specific compositions, emphasizing the utility of fine-scale monitoring in understanding microbial diversity and potential functionalities [17, 50].

The lower α-diversity of roots compared to other compartments highlights a selective barrier at the rhizoplane, influencing bacterial entry and potentially reducing diversity within root microbiomes (Supp. Figure S8). The comparative analysis showing lower richness in roots and leaves, similar to patterns observed in *A. thaliana*, suggests similarities in endosphere microbial diversity across plant species [15]. This observation aligns with previous studies which have demonstrated that the structure of microbial communities is influenced by the plant compartment, the location sampled, and the identity of the host species [51].

The higher diversity in compartments surrounding *A. marina* roots and leaves implies greater variability in these external compartments. The role of the rhizoplane emerges as a critical regulator controlling microbial access to roots, impacting overall microbial diversity in root-related compartments. These results highlight the importance of precise root-associated microbiome analyses and highlight the intricate relationships shaping microbial communities within mangrove ecosystems, crucial for understanding their resilience and ecological roles [52–54].

Common bacterial taxa were identified in the studied ecosystem, revealing *Rhizobeacea*,* Halomonadaceae*,* Commamonadaceae*, and *Bacillaceae* as representatives of the mangrove ecosystem (Fig. 2C). The presence of sulfide-reducing bacteria in the rhizosphere underscores their critical ecological role as anaerobic decomposers in the mangrove environment [45]. These microbes facilitate the breakdown of organic sulfur compounds, a process that not only detoxifies sulfide—a common byproduct of anaerobic respiration—but also releases essential nutrients, such as iron and phosphorus. These nutrients are crucial for the growth and metabolic functions of various organisms within the mangrove ecosystem, supporting both microbial and plant health [43]. Moreover, the adaptability of these microbial communities reflects their sophisticated mechanisms for coping with the unique and often harsh conditions of mangrove habitats, such as fluctuating salinity, oxygen limitation, and high organic matter content. The taxonomic composition of *A. marina*’s bacterial community further emphasizes the role of these microbes in enhancing the resilience of mangroves to environmental stressors [44]. This microbial resilience likely contributes to the stability and productivity of mangrove ecosystems, aiding in nutrient cycling, stress tolerance, and overall ecosystem sustainability. These findings highlight the intricate relationships between mangroves and their associated microbial communities, suggesting that the functional capabilities of these bacteria are integral to the persistence of mangroves in challenging coastal environments [46].

Our study supports the hypothesis that environmental factors such as salinity and nutrient availability are primary drivers of microbial community structure and function in the rhizosphere. These findings are consistent with other research showing that the functional profiles of microbial communities are significantly influenced by local environmental conditions. On the other hand, the endophytic bacterial community structure is shaped principally via host plant-based needs [6, 27].

#### Bacterial diversity and compartment specificity

Beta diversity analysis using Principal Coordinates Analysis (PCoA) and Non-metric Multidimensional Scaling (NMDS) demonstrated a clear separation between microbial communities of different compartments. The clustering of samples from bulk soil and rhizosphere (Fig. 2D and Table S6), and their distinction from root and leaf samples, underscores the compartment-specific microbial assemblages. This is consistent with previous studies showing that plant compartments harbour distinct microbial communities influenced by factors such as nutrient availability, root exudates, and environmental conditions [55, 56]. Our findings revealed distinct bacterial diversity in the rhizosphere, likely due to the selective influences of the roots. The roots appear to create a unique microenvironment through their exudates, which selectively attract and sustain specific bacterial communities. This selective pressure results in a rhizosphere microbial community that differs significantly from that of the bulk soil, highlighting the important role of plant-microbe interactions in shaping microbial diversity and function in the rhizosphere. This selective process might attract specific bacterial types while reducing the abundance of other groups within the rhizosphere environment [57]. Root exudates should select bacterial groups in the rhizosphere. Hence, changes in the exudates at different stages of *A. marina* growth could alter the composition and abundance of bacterial groups. It was reported earlier that mangrove roots reduced phenol oxidase but increased hydrolysates, which are related to the mineralization of important nutrients such as N and P [56] in the rhizosphere and rhizoplane [58]. In line with these data, PCoA results exhibited a distribution pattern of the microbial communities from different compartments showing closer relative distances except for rhizosphere and leaves. On the contrary, there was a clear difference between the microbiome of leaves and rhizoplane and the different microbiomes of root, bulk soil and rhizosphere (Fig. 2D). The NMDS data indicated differences among the compartments which was further supported by the MRPP test (Table S5) and ANOSIM, *P* < 0.01 (Fig. 2E and Figure S8). The distinct microbial communities observed in the root and leaf endospheres likely reflect the specialized roles these microbes play in plant health, such as promoting growth, enhancing salinity tolerance, or protecting against pathogens. In contrast, the more diverse and variable communities in the bulk soil and rhizosphere suggest a broader range of ecological functions, including nutrient cycling and organic matter decomposition, influenced by environmental factors like salinity and soil texture [59, 60].

### Mangrove ecosystems: biomarker taxa analysis

Understanding microbial community dynamics is crucial for grasping how Mangrove ecosystem’s function. Mangrove microbial communities constantly change due to environmental factors which ultimately influence ecosystem functioning and resilience. Through detailed analyses such as identifying biomarker taxa and conducting LDA assessments, we aimed to uncover the mechanisms driving these changes. All LDA scores exceeded 4.0, indicating reliability in identifying biomarker taxa (Fig. 3A). The number of biomarker taxa decreased with increasing diversity. Dynamic compartments like the rhizosphere and rhizoplane had fewer unique taxa (6 taxa with LDA > 4.0) compared to other compartments (twice as many with LDA ≥ 4.5). Notably, Acidimicrobiia and Rhodothermia were abundant in bulk soil. These bacteria are known for producing organic acids that are beneficial for plant growth. Enriched taxa in roots included Burkholderiales, Rhizobiales and Pseudomonadales, contributing to nutrient cycling and nitrogen assimilation. Lower LDA scores in root biomarkers may stem from host-specificity and plant developmental stages. Leaves exhibited enrichment of *Salinisphaera*, *Lactobacellales* and *Pseudomonas*, indicating adaptation to salinity and nutrient limitations. Bacillus and Desulfobacterota were prominent in the rhizosphere, contributing to nutrient cycling and adaptation to harsh environments. These bacteria could facilitate the growth of *A. marina* under limited nutrient conditions such as nitrogen and phosphorus, as proved earlier [11].

In the rhizoplane, *Ralstonia solanacearum* and Rhizoboiales were significant biomarkers. Those enriched taxa usually denitrify oxidized N compounds such as Nitrate NO^− 3^ (or Nitrite NO^− 2^) into atmospheric N_2_ under low oxygen or aerobic conditions. Nitrate depletion causes an increased N demand, which may lead to increased competition between plants and soil microorganisms [61]. Beneficial bacteria like Bacillus in soils may suppress pathogenic bacteria from entering roots. Significant differences were observed between roots and leaves in the abundance of certain taxa, highlighting compartment-specific microbiome dynamics.

In addition, the biomarkers in the root with lower LDA scores might be due to the host-specificity of roots and leaves [62], which is highly dynamic and mainly influenced by the plant developmental stage compared to the rhizosphere and bulk soil-related communities [63]. Furthermore, the highly enriched taxa in leaves were *Salinisphaera*, *Lactobacellales* and *Pseudomonas* (Fig. 3A and B-2). LEfSe showed that *Bacillus* and Desulfobacterota are the most significant biomarkers and the highest taxon level in the rhizosphere. Desulfobacterota reduces sulfate to sulfite the main limiting elements of the *A. marina* growth, along with C and N [64]. Some Desulfobacteria were found in the sediments of mangroves and aerobically autotroph plants at 25–30 cm depth. This bacterial group is considered as a keystone taxon for the mangrove microbiome [65, 66]. *Bacillus* is considered a healthy mangrove soil indicator, and their ubiquity might be due to the ability to form endospores to adapt to harsh environments. Bacillus also enhances the presence of ammonia-oxidizing bacteria (AOB) in the soil, such as *Nitrosospira* and *Nitrosomonas* [67], and the Dissimilatory Nitrate Reduction to Ammonia (DNRA) [50]. In summary, the data on the microbiome of *A. marina* within the mangrove ecosystem aligns with the broader understanding of mangrove microbiomes worldwide. These similarities emphasize the importance of microbial communities in shaping mangrove ecosystem dynamics, supporting plant health, nutrient cycling, and adaptation to environmental stressors e.g. salt stress, and heavy mental contamination, thus contributing to the sustainability and resilience of mangrove ecosystems.

#### Novel insights and applications

The comprehensive collection of 256 bacterial strains isolated from *A. marina* represents a highly valuable resource for agriculture, industry, and ecological restoration. Their exceptional halotolerance and thermophilic capabilities make them ideal candidates for biotechnological applications in extreme environments, ensuring robust performance under stress conditions [46]. The uniqueness of this collection lies in its blend of terrestrial and marine bacteria, adapted to thrive in the mangrove ecosystem’s dynamic intertidal environment. This dual adaptability allows for the exploration of microbial functions across different ecological niches, offering insights into their versatile metabolic capabilities.

The dominance of genera such as Bacillus, Pseudomonas, and Halomonas aligns with their known capabilities for plant growth promotion and bioremediation. The functional assays further confirmed the ability of these isolates to produce plant hormones, degrade organic pollutants, and enhance nutrient availability, corroborating their ecological roles in the mangrove microbiome. Moreover, the bacterial community composition varied across different compartments, encompassing over 70 bacterial phyla (Fig. 5 and Figure S12).

In comparison to the culture-independent microbiome data, we succeeded in recovering many the bacterial groups with recovery rate of 56%, including Proteobacteria, Firmicutes, Bacteroidota, and Actinobacteriota (Fig. 6A, Figure S7) except for Desulfobacterota, Bdellovibrionota, and Acidobacteriota. The absence of these phyla ( Figure S14) is due to the difficulty in cultivating those bacteria [68, 69]. The relative abundance profiles of the major cultural bacterial groups differ in bulk soil and other mangrove compartments. Additionally, the complexity decreases from the rhizosphere to the roots. Cultural as non-cultural approaches showed a high abundance of Proteobacteria, mainly *Microbulbifer* (35%) in bulk soil, *Halomonas* (24%) and *Vibrio* (15%) in the rhizosphere, and *Larsenimonas* (70%) in the roots (Figure S12) indicating a selection pressure of functional traits, mainly siderophores and pectinase producers with 85% positive strains, respectively (Fig. 5; Table 1). Those bacteria might play a role in increasing the amount of Fe needed for healthy plant growth and catalysing the degradation of pectin polymers in the plant cell walls [70], facilitating bacterial root colonization.

Secondly, Firmicutes, mainly *Halobacillus* in bulk soil (18%) and Rhizoplane (5%), while *Bacillus* (5%) in the rhizosphere (Figure. S15), were also found to largely produce siderophores, pectinases, and proteases (Dataset). Therefore, these genera may facilitate the biodegradation of organic material in soil. Thirdly, Actinobacteriota, represented by *Isoptericola* in bulk soil (18%), 4% in rhizosphere, 2% in rhizoplane, and 4% in roots, were less abundant in the rhizosphere and rhizoplane (Figure S12- B and C). These bacteria are involved in phosphate uptake and the degradation of cellulose, hemicellulose and chitin [6, 71]. Noteworthy, it has been revealed that ACC deaminase-producing strains of *Isoptericola* and *Bacillus* stimulated the growth of the host plant and influenced flavonoid accumulation [72], which is known for stress alleviation [73]. The least abundant group were Bacteroidota, such as *Neptunitalea*, mainly found in the rhizosphere and rhizoplane (Dataset). This group of bacteria is known to play a pivotal role in denitrification and organic carbon degradation [71]. Proteobacteria dominated the Mangrove bacterial collection and exhibited predominantly positive traits for siderophore and pectinase hydrolase (~ 85% for both traits). However, other Plant Growth-Promoting (PGP) traits such as proteases (38.3%), cellulases (22.3%), and phosphate solubilization (3.5%) were notably lower.

#### Showcase and application prospective

Using the culture-dependent mangrove library, we aimed to harness the potential of beneficial mangrove-associated bacteria to combat salt stress tolerance of plants. To achieve this, we subjected Arabidopsis to 100 mM NaCl, a condition known to strongly inhibit plant growth. Out of a total of 77 isolates selected for testing on *A. thaliana* under normal or salt-stress conditions, 42 showed significant enhancement of growth, demonstrating a beneficial increase of more than 20% compared to the control in the presence of ½ MS with 100mM NaCl (Table S7).

Eleven isolates (AK031, AK116, AK157, AK164, AK171, AK181, AK185, AK223 and AK255) enhanced the FW under normal or salt conditions (Dataset and Fig. 7). Previous studies have shown that bacteria isolated from mangrove sediments, such as *Halomonas*, can help alleviate plant stress tolerance under salinity and heavy metal stress conditions. This suggests that mangroves and associated bacteria may have potential applications in sustainable trials for phytomanagement and bioremediation [47].

The aquatic *Pseudoalteromonas* has been applied as coral probiotics, protecting the host against pathogens and heat stress. This might be due to biosynthetic gene clusters (BGCs) encoding ectoine, T2SS, and T8SS [74]. The Actinobacteriotal strains AK157 (*D. activiva)*, AK164 [42], AK225 (*I. chiayiensis*) can solubilize phosphate and carbohydrate hydrolysis by extracellular enzyme production, such as degradation of cellulose, hemicellulose, and chitin [71], which may contribute to both plant growth and salt tolerance. The beneficial effect of *T. mobilis* AK171 [75] and AK185 strains might be due to the ability of this group of bacteria to produce an antibiotic agent called tropodithietic acid (TDA), which enhances microalgal health by killing pathogens [76].

In the other part of the experiment, the 5 days old seedlings were transferred to a submerged disc media to mimic the waterlogging and salinity stress (100mM) to further shorten the list of our candidates as stress alleviators of *A. thaliana*. By this method, only four out of the 11 previous candidates significantly increased the fresh weight of inoculated *(A) thaliana* seedlings compared to non-inoculated plants (Table 12; Figs. 43, 44, 45 and 46). Those strains included Firmicutes (AK031, *(B) seohaeanensis*; AK116, *H. locisalis*), an actinomycete (AK164), as well as two Proteobacteria (*Roseobacter* sp. AK171, *T. mobilis*, *Pseudoalteromonas* AK181 and AK223 *P. azotoformans*). *B. pumilus* FAB10 proved to possess high salt tolerance and PGP traits and considered as a PGPB of salinity-treated wheat (*Triticum aestivum*) seeds [77]. Research has shown that halotolerant bacteria such as *Hallobacillus* sp. SL3 and *Bacillus halodenitrificans* PU62, both isolated from saline environments, possess significant potential to enhance plant growth under conditions of saline stress [78] These bacteria exert their beneficial effects primarily through the production of indole-3-acetic acid (IAA) and siderophores. The promotion of the growth in the *Isoptericola* sp. AK164 -inoculated *A. thaliana* plants in both normal and stress conditions might be due to the ability to produce ACC deaminase to alleviate stress [42, 72].PGP trait analysis showed that AK171 could produce siderophores (iron chelators), pectinase, and IAA. Iron chelators produced by this strain help lower ethylene levels in developing plant roots, resulting in increased root growth [79, 80]. Pectinase is also essential for endophytes to facilitate the entry into plant tissues [81], and both pectinase and cellulase activity are positively correlated with salt and osmotic tolerance, indicating the importance of these properties in survival and colonization [82]. Another indirect promotion of plant growth by AK171 might stem from its ability to produce siderophores, as revealed for siderophore-producing *P. putida*, in which a greater translocation of iron from roots to the grain emphasizes the importance of siderophores in the beneficial bacterial-rice interaction [83].

Interestingly, despite the presence of a large number of bacteria with potential growth promotion, the PGP test strain scoring did not strongly correlate with plant growth promotion or salinity stress alleviation. This discrepancy might stem from several factors, including insufficient growth enhancement or stress alleviation, potential incompatibility in plant colonization, or a lack of host genetic affiliation between PGP bacteria and the plant host. Studies suggest that while gene clusters indicating potential PGP functions are informative, they do not necessarily predict a strain’s capability to promote plant growth under abiotic stress conditions [9, 42, 84, 85]. It becomes increasingly clear that the recruitment of endophytic bacteria by plants is determined not solely by the taxonomy but more significantly by the functional bacterial traits required by the plants, such as salt tolerance in our case. This understanding underscores the importance of selecting microbial strains based on their functional capabilities rather than just their genetic potential. The observed PGP traits and stress tolerance abilities of these isolated bacteria highlight their potential contribution to plant growth promotion and their adaptability to diverse environmental conditions, including saline, drought, and thermophilic ecosystems.

Combining in-plant screening with PGP trait assessment could significantly enhance the success of selecting the right candidates. By integrating these approaches, it is possible to identify bacterial strains that not only possess desirable PGP traits in vitro but also demonstrate effective plant growth promotion and stress alleviation in real-world conditions. This dual screening strategy can ensure the selection of the most compatible and effective microbial inoculants, maximizing their potential benefits for agricultural and environmental applications.

## Conclusion

Our study of the *Avicennia marina* microbiome uncovers the intricate relationships between mangrove plants and their associated microbial communities, characterized by distinct assemblages and functional traits across various plant compartments. These traits reflect adaptive strategies for thriving in saline environments. The diverse collection of bacteria, particularly from the rhizosphere, rhizoplane, and endophytes, showcases significant plant growth-promoting (PGP) traits and stress tolerance capabilities, underscoring the importance of functional attributes, such as salt tolerance, in bacterial recruitment over taxonomic affiliation. Notably, our findings are supported by research demonstrating that mangrove-derived microbes have been successfully applied to enhance growth and stress resilience in various non-mangrove plant systems. This success underscores the potential of these microbes to serve as effective biofertilizers and agents for bioremediation in diverse agricultural contexts. As we continue to explore these applications, extensive field trials and scalability studies will be essential to fully harness the power of the mangrove microbiome for broader agricultural and ecological benefits.

## Electronic supplementary material

Below is the link to the electronic supplementary material.

Supplementary Material 1

Supplementary Material 2

## Data Availability

The 16 S rRNA gene sequences of the bacterial isolates in this study have been deposited in the GenBank database and are accessible under accession numbers (OR447762 - OR448017). 16 S microbiome sequence data generated in this study are available at NCBI under the BioProject ID PRJNA1063583.

## References

[1] United Nations DoEaSA, Population Division. World Population Prospects 2022: Summary of Results. *UN DESA/POP/2022/TR/NO 3* 2022.

[2] Qadir M, Quillérou E, Nangia V, Murtaza G, Singh M, Thomas RJ, Drechsel P, Noble AD. Economics of salt-induced land degradation and restoration. Nat Resour Forum. 2014;38(4):282–95.

[3] Kearl J, McNary C, Lowman JS, Mei C, Aanderud ZT, Smith ST, West J, Colton E, Hamson M. BL. N: salt-tolerant halophyte rhizosphere Bacteria stimulate growth of Alfalfa in Salty Soil. Front Microbiol 2019, 10(1849). 10.3389/fmicb.2019.01849 PMC6702273 31474952

[4] Kumar A, Singh S, Gaurav AK, Srivastava S, Verma JP. Plant growth-promoting Bacteria: Biological Tools for the mitigation of salinity stress in plants. Front Microbiol 2020, 11. 10.3389/fmicb.2020.01216 PMC7358356 32733391

[5] Saad MM, Eida AA, Hirt H. Tailoring plant-associated microbial inoculants in agriculture: a roadmap for successful application. J Exp Bot. 2020;71(13):3878–901. 32157287 10.1093/jxb/eraa111 PMC7450670

[6] Eida AA, Ziegler M, Lafi FF, Michell CT, Voolstra CR, Hirt H, Saad MM. Desert plant bacteria reveal host influence and beneficial plant growth properties. PLoS ONE. 2018;13(12):e0208223. 30540793 10.1371/journal.pone.0208223 PMC6291088

[7] Boukhatem ZF, Merabet C, Tsaki H. Plant Growth promoting Actinobacteria, the most promising candidates as Bioinoculants? Front Agron. 2022;4(March):1–19.

[8] de Zelicourt A, Synek L, Saad MM, Alzubaidy H, Jalal R, Xie Y, Andres-Barrao C, Rolli E, Guerard F, Mariappan KG, et al. Ethylene induced plant stress tolerance by Enterobacter sp. SA187 is mediated by 2-keto-4-methylthiobutyric acid production. PLoS Genet. 2018;14(3):e1007273. 29554117 10.1371/journal.pgen.1007273 PMC5875868

[9] Alwutayd KM, Rawat AA, Sheikh AH, Almeida-Trapp M, Veluchamy A, Jalal R, Karampelias M, Froehlich K, Alzaed W, Tabassum N et al. Microbe-induced drought tolerance by ABA-mediated root architecture and epigenetic reprogramming. EMBO Rep 2023:e56754. 10.15252/embr.202256754 PMC10398642 37278352

[10] Asaeda T, Barnuevo A. Oxidative stress as an indicator of niche-width preference of mangrove Rhizophora Stylosa. For Ecol Manag. 2019;432(September 2018):73–82.

[11] Almahasheer H, Duarte CM, Irigoien X. Nutrient limitation in central red sea mangroves. Front Mar Sci. 2016;3(DEC):1–14.

[12] Nizam A, Meera SP, Kumar A. Genetic and molecular mechanisms underlying mangrove adaptations to intertidal environments. iScience. 2022;25(1):103547–103547. 34988398 10.1016/j.isci.2021.103547 PMC8693430

[13] Alhassan AB, Aljahdali MO. Nutrient and physicochemical properties as potential causes of stress in mangroves of the central Red Sea. PLoS ONE. 2021;16(12 December):1–19. 10.1371/journal.pone.0261620 PMC8700010 34941948

[14] Mai Z, Ye M, Wang Y, Foong SY, Wang L, Sun F, Cheng H. Characteristics of Microbial community and function with the succession of mangroves. Front Microbiol 2021, 12. 10.3389/fmicb.2021.764974 PMC8689078 34950118

[15] Zhuang W, Yu X, Hu R, Luo Z, Liu X, Zheng X, Xiao F, Peng Y, He Q, Tian Y et al. Diversity, function and assembly of mangrove root-associated microbial communities at a continuous fine-scale. Npj Biofilms Microbiomes 2020, 6(1). 10.1038/s41522-020-00164-6 PMC7665043 33184266

[16] Kimbrel JA, Ballor N, Wu YW, David MM, Hazen TC, Simmons BA, Singer SW, Jansson JK. Microbial community structure and functional potential along a hypersaline gradient. Front Microbiol. 2018;9(JUL):1–15. 30042744 10.3389/fmicb.2018.01492 PMC6048260

[17] Gómez-Godínez LJ, Fernandez-Valverde SL, Martinez Romero JC, Martínez-Romero E. Metatranscriptomics and nitrogen fixation from the rhizoplane of maize plantlets inoculated with a group of PGPRs. Syst Appl Microbiol. 2019;42(4):517–25. 31176475 10.1016/j.syapm.2019.05.003

[18] Simon M, Scheuner C, Meier-Kolthoff JP, Brinkhoff T, Wagner-Döbler I, Ulbrich M, Klenk H-P, Schomburg D, Petersen J, Göker M. Phylogenomics of Rhodobacteraceae reveals evolutionary adaptation to marine and non-marine habitats. ISME J. 2017;11:1483–99. 28106881 10.1038/ismej.2016.198 PMC5437341

[19] Newton RJ, Griffin LE, Bowles KM, Meile C, Gifford S, Givens CE, Howard EC, King E, Oakley CA, Reisch CR, et al. Genome characteristics of a generalist marine bacterial lineage. ISME J. 2010;4(6):784–98. 20072162 10.1038/ismej.2009.150

[20] Yoolong S, Kruasuwan W, Thanh Phạm HT, Jaemsaeng R, Jantasuriyarat C, Thamchaipenet A. Modulation of salt tolerance in Thai jasmine rice (Oryza sativa L. Cv. KDML105) by Streptomyces venezuelae ATCC 10712 expressing ACC deaminase. Sci Rep. 2019;9(1):1–10. 30718781 10.1038/s41598-018-37987-5 PMC6361907

[21] Haque MM, Biswas MS, Mosharaf MK, Haque MA, Islam MS, Nahar K, Islam MM, Shozib HB, Islam MM, Ferdous EE. Halotolerant biofilm-producing rhizobacteria mitigate seawater-induced salt stress and promote growth of tomato. Sci Rep. 2022;12(1):1–22. 35379908 10.1038/s41598-022-09519-9 PMC8980105

[22] Ali S, Kim WC. Plant growth promotion under water: Decrease of waterlogging-induced ACC and ethylene levels by ACC deaminase-producing bacteria. *Frontiers in Microbiology* 2018, 9(MAY). 10.3389/fmicb.2018.01096 PMC5981179 29887854

[23] Akaji Y, Inoue T, Taniguchi T, Baba S. Arbuscular mycorrhizal fungal communities of a mangrove forest along a salinity gradient on Iriomote Island. Plant Soil. 2022;472(1):145–59.

[24] Lee NLY, Huang D, Quek ZBR, Lee JN, Wainwright BJ. Mangrove-Associated Fungal communities are differentiated by Geographic location and host structure. Front Microbiol 2019, 10. 10.3389/fmicb.2019.02456 PMC6831645 31736902

[25] Schau H-P: J. F. MacFaddin, Media for Isolation - Cultivation - Identification - Maintenance of Medical Bacteria, Volume, XI + 929 I. S., 163 Abb., 94 Tab. Baltimore, London 1985. Williams and Wilkins. $ 90.00. ISBN: 0-683-05316-7. *Journal of Basic Microbiology* 1986, 26(4):240–240.

[26] Pommerville JC. Fundamentals of microbiology. Jones & Bartlett; 2013.

[27] Eida AA, Alzubaidy HS, de Zélicourt A, Synek L, Alsharif W, Lafi FF, Hirt H, Saad MM. Phylogenetically diverse endophytic bacteria from desert plants induce transcriptional changes of tissue-specific ion transporters and salinity stress in Arabidopsis thaliana. Plant Sci. 2019;280:228–40. 30824001 10.1016/j.plantsci.2018.12.002

[28] Asha B, Palaniswamy M. Optimization of alkaline protease production by Bacillus cereus FT 1 isolated from soil. J Appl Pharm Sci. 2018;8(2):119–27.

[29] De Vuyst L, Fr´ F, Fréd´ F, Leroy F. Functional role of yeasts, lactic acid bacteria and acetic acid bacteria in cocoa fermentation processes. FEMS Microbiol Rev. 2020;014:432–53. 10.1093/femsre/fuaa014 32420601

[30] Bolyen E, Rideout JR, Dillon MR, Bokulich NA, Abnet CC, Al-Ghalith GA, Alexander H, Alm EJ, Arumugam M, Asnicar F, et al. Reproducible, interactive, scalable and extensible microbiome data science using QIIME 2. Nat Biotechnol. 2019;37(8):852–7. 31341288 10.1038/s41587-019-0209-9 PMC7015180

[31] Bokulich NA, Kaehler BD, Rideout JR, Dillon M, Bolyen E, Knight R, Huttley GA, Gregory Caporaso J. Optimizing taxonomic classification of marker-gene amplicon sequences with QIIME 2’s q2-feature-classifier plugin. Microbiome. 2018;6(1):90. 29773078 10.1186/s40168-018-0470-z PMC5956843

[32] Li B, Zhang X, Guo F, Wu W, Zhang T. Characterization of tetracycline resistant bacterial community in saline activated sludge using batch stress incubation with high-throughput sequencing analysis. Water Res. 2013;47(13):4207–16. 23764571 10.1016/j.watres.2013.04.021

[33] Lozupone C, Knight R. UniFrac: a new phylogenetic method for comparing microbial communities. Appl Environ Microbiol. 2005;71(12):8228–35. 16332807 10.1128/AEM.71.12.8228-8235.2005 PMC1317376

[34] Minchin PR. An evaluation of the relative robustness of techniques for ecological ordination. *Vegetatio* 1978, 69, pages89–107 (1987).

[35] Kruskal JB. Nonmetric multidimensional scaling: a numerical method. Psychometrika. 1964;29:115–29.

[36] Chapman MG, Underwood AJ. Ecological patterns in multivariate assemblages: information and interpretation of negative values in ANOSIM tests. Mar Ecol Prog Ser. 1999;180:257–65.

[37] Stat M, Pochon X, Franklin EC, Bruno JF, Casey KS, Selig ER, Gates RD. The distribution of the thermally tolerant symbiont lineage (Symbiodinium clade D) in corals from Hawaii: correlations with host and the history of ocean thermal stress. Ecol Evol. 2013;3(5):1317–29. 23762518 10.1002/ece3.556 PMC3678486

[38] Segata N, Izard J, Waldron L, Gevers D, Miropolsky L, Garrett WS, Huttenhower C. Metagenomic biomarker discovery and explanation. Genome Biol. 2011;12:R60. 21702898 10.1186/gb-2011-12-6-r60 PMC3218848

[39] Douglas GM, Maffei VJ, Zaneveld JR, Yurgel SN, Brown JR, Taylor CM, Huttenhower C, Langille MGI. PICRUSt2 for prediction of metagenome functions. Nat Biotechnol. 2020;38(6):685–8. 32483366 10.1038/s41587-020-0548-6 PMC7365738

[40] Galperin MY, Wolf YI, Makarova KS, Alvarez RV, Landsman D, Koonin EV. COG database update: focus on microbial diversity, model organisms, and widespread pathogens. Nucleic Acids Res. 2021;49(D1):D274–81. 33167031 10.1093/nar/gkaa1018 PMC7778934

[41] Bastian M, Heymann S, Jacomy M. Gephi: An Open Source Software for Exploring and Manipulating Networks. 2014.

[42] Alghamdi AK, Parween S, Hirt H, Saad MM. Complete genome sequence analysis of plant growth-promoting bacterium, Isoptericola sp. AK164 isolated from the rhizosphere of Avicennia marina growing at the Red Sea coast. Arch Microbiol. 2023;205(9):307. 37580455 10.1007/s00203-023-03654-1 PMC10425560

[43] Thomson T, Fusi M, Bennett-Smith MF, Prinz N, Aylagas E, Carvalho S, Lovelock CE, Jones BH, Ellis JI. Contrasting effects of Local Environmental and Biogeographic factors on the composition and structure of bacterial communities in Arid Monospecific Mangrove soils. Microbiol Spectr 2022, 10(1). 10.1128/spectrum.00903-21 PMC8729789 34985338

[44] Holguin G, Vazquez P, Bashan Y. The role of sediment microorganisms in the productivity, conservation, and rehabilitation of mangrove ecosystems: an overview. Biol Fertil Soils. 2001;33(4):265–78.

[45] Bernardino AF, Sanders CJ, Bissoli LB, Gomes LEO, Kauffman JB, Ferreira TO. Land use impacts on benthic bioturbation potential and carbon burial in Brazilian mangrove ecosystems. Limnol Oceanogr. 2020;65(10):2366–76.

[46] Thatoi H, Behera BC, Mishra RR, Dutta SK. Biodiversity and biotechnological potential of microorganisms from mangrove ecosystems: a review. Ann Microbiol. 2013;63(1):1–19.

[47] Al-Solaimani SG, Abohassan RA, Alamri DA, Yang X, Rinklebe J, Shaheen SM. Assessing the risk of toxic metals contamination and phytoremediation potential of mangrove in three coastal sites along the Red Sea. *Marine Pollution Bulletin* 2022, 176(July 2021). 10.1016/j.marpolbul.2022.113412 35168071

[48] Mhete M, Eze PN, Rahube TO, Akinyemi FO. Soil properties influence bacterial abundance and diversity under different land-use regimes in semi-arid environments. Sci Afr 2020, 7.

[49] Aguilar-Paredes A, Valdés G, Araneda N, Valdebenito E, Hansen F, Nuti M. Microbial Community in the composting process and its positive impact on the Soil Biota in Sustainable Agriculture. Agronomy-Basel 2023, 13(2).

[50] Sun B, Bai Z, Bao L, Xue L, Zhang S, Wei Y, Zhang Z, Zhuang G, Zhuang X. Bacillus subtilis biofertilizer mitigating agricultural ammonia emission and shifting soil nitrogen cycling microbiomes. Environ Int. 2020;144(April):105989–105989. 32739514 10.1016/j.envint.2020.105989

[51] Wainwright BJ, Millar T, Bowen L, Semon L, Hickman KJE, Lee JN, Yeo ZY, Zahn G. The core mangrove microbiome reveals shared taxa potentially involved in nutrient cycling and promoting host survival. Environ Microbiome. 2023;18(1):47. 37264467 10.1186/s40793-023-00499-5 PMC10236742

[52] Edwards J, Johnson C, Santos-Medellín C, Lurie E, Podishetty NK, Bhatnagar S, Eisen JA, Sundaresan V, Jeffery LD. Structure, variation, and assembly of the root-associated microbiomes of rice. Proc Natl Acad Sci USA. 2015;112(8):E911–20. 25605935 10.1073/pnas.1414592112 PMC4345613

[53] Lundberg DS, Lebeis SL, Paredes SH, Yourstone S, Gehring J, Malfatti S, Tremblay J, Engelbrektson A, Kunin V, Del Rio TG, et al. Defining the core Arabidopsis thaliana root microbiome. Nature. 2013;501(7468 SUPPL):86–90. 10.1038/nature11237 PMC4074413 22859206

[54] Bulgarelli D, Rott M, Schlaeppi K, van Ver Loren E, Ahmadinejad N, Assenza F, Rauf P, Huettel B, Reinhardt R, Schmelzer E, et al. Revealing structure and assembly cues for Arabidopsis root-inhabiting bacterial microbiota. Nature. 2012;488(7409):91–5. 22859207 10.1038/nature11336

[55] Lin XHBLL, et al. Mangrove Sediment Microbiome: adaptive microbial assemblages and their routed biogeochemical processes in Yunxiao Mangrove National Nature Reserve, China. Microb Ecol. 2019;78(1):57–69. 30284602 10.1007/s00248-018-1261-6

[56] Luo L, Wu R, Gu JD, Zhang J, Deng S, Zhang Y, Wang L, He Y. Influence of mangrove roots on microbial abundance and ecoenzyme activity in sediments of a subtropical coastal mangrove ecosystem. Int Biodeterior Biodegradation. 2018;132(May):10–7.

[57] DeAngelis KM, Brodie EL, DeSantis TZ, Andersen GL, Lindow SE, Firestone MK. Selective progressive response of soil microbial community to wild oat roots. ISME J. 2009;3(2):168–78. 19005498 10.1038/ismej.2008.103

[58] Zhang LH, Song LP, Xu G, Chen P, Sun JN, Shao HB. Seasonal Dynamics of Rhizosphere Soil Microbial Abundances and enzyme activities under different vegetation types in the Coastal Zone, Shandong, China. Clean - Soil Air Water. 2014;42(8):1115–20.

[59] Sun D, Huang Y, Wang Z, Tang X, Ye W, Cao H, Shen H. Soil microbial community structure, function and network along a mangrove forest restoration chronosequence. Sci Total Environ. 2024;913:169704. 38163592 10.1016/j.scitotenv.2023.169704

[60] de Carvalho FM, Laux M, Ciapina LP, Gerber AL, Guimarães APC, Kloh VP, Apolinário M, Paes JES, Jonck CR, de Vasconcelos ATR. Finding microbial composition and biological processes as predictive signature to access the ongoing status of mangrove preservation. Int Microbiol 2024. 10.1007/s10123-024-00492-z PMC11452435 38388811

[61] Hu S, Firestone MK, Chapin FS, Hu SFMK, Chapin FS. Soil microbial feedbacks to atmospheric CO2 enrichment. Trends Ecol Evol. 1999;14(11):433–7. 10511719 10.1016/s0169-5347(99)01682-1

[62] Suryanarayanan TS, Kumaresan V, Johnson JA. Foliar fungal endophytes from two species of the mangrove Rhizophora. Can J Microbiol. 1998;44(10):1003–6.

[63] Usyskin-Tonne A, Hadar Y, Yermiyahu U, Minz D. Elevated CO2 has a significant impact on denitrifying bacterial community in wheat roots. Soil Biol Biochem. 2020;142(December 2019):107697–107697.

[64] Lin YT, Lin YF, Tsai IJ, Chang EH, Jien SH, Lin YJ, Chiu CY. Structure and diversity of Soil Bacterial communities in Offshore Islands. Sci Rep. 2019;9(1):1–9. 30894580 10.1038/s41598-019-41170-9 PMC6426884

[65] Liu Y, Zhou Z, Yang Y, Li M. Genome-scale Co-occurrence and Co-transcription Networks Reveal Key Microbial Taxa in Mangrove Sediments. 2020:1–13.

[66] Du H, Pan J, Zou D, Huang Y, Liu Y, Li M. Microbial active functional modules derived from network analysis and metabolic interactions decipher the complex microbiome assembly in mangrove sediments. Microbiome. 2022;10(1):224. 36510268 10.1186/s40168-022-01421-w PMC9746113

[67] Ai C, Liang G, Sun J, Wang X, He P, Zhou W. Different roles of rhizosphere effect and long-term fertilization in the activity and community structure of ammonia oxidizers in a calcareous fluvo-aquic soil. Soil Biol Biochem. 2013;57:30–42.

[68] Ansari FA, Ahmad I, Pichtel J. Growth stimulation and alleviation of salinity stress to wheat by the biofilm forming Bacillus pumilus strain FAB10. Appl Soil Ecol. 2019;143(December 2018):45–54.

[69] Waite DW, Chuvochina M, Pelikan C, Parks DH, Yilmaz P, Wagner M, Loy A, Naganuma T, Nakai R, Whitman WB, et al. Proposal to reclassify the proteobacterial classes deltaproteobacteria and oligoflexia, and the phylum thermodesulfobacteria into four phyla reflecting major functional capabilities. Int J Syst Evol MicroBiol. 2020;70(11):5972–6016. 33151140 10.1099/ijsem.0.004213

[70] Kavuthodi B, Sebastian D. Review on bacterial production of alkaline pectinase with special emphasis on Bacillus species. Bioscience Biotechnol Res Commun. 2018;11(1):18–30.

[71] Yilmaz P, Yarza P, Rapp JZ, Glöckner FO. Expanding the world of marine bacterial and archaeal clades. Front Microbiol. 2016;6(JAN):1–29. 10.3389/fmicb.2015.01524 PMC4705458 26779174

[72] Qin S, Zhang YJ, Yuan B, Xu PY, Xing K, Wang J, Jiang JH. Isolation of ACC deaminase-producing habitat-adapted symbiotic bacteria associated with halophyte Limonium sinense (Girard) Kuntze and evaluating their plant growth-promoting activity under salt stress. Plant Soil. 2014;374(1–2):753–66.

[73] Winkel-Shirley B. Biosynthesis of flavonoids and effects of stress. Curr Opin Plant Biol. 2002;5(3):218–23. 11960739 10.1016/s1369-5266(02)00256-x

[74] Rosado PM, Leite DCA, Duarte GAS, Chaloub RM, Jospin G, Nunes da Rocha U, Dini-Andreote JPS, Eisen F, Bourne JA. Marine probiotics: increasing coral resistance to bleaching through microbiome manipulation. ISME J. 2019;13(4):921–36. 30518818 10.1038/s41396-018-0323-6 PMC6461899

[75] Alghamdi AK, Parween S, Hirt H, Saad MM. Unraveling the genomic secrets of Tritonibacter mobilis AK171: a plant growth-promoting bacterium isolated from Avicennia marina. BMC Genomics. 2024;25(1):672. 38969999 10.1186/s12864-024-10555-0 PMC11225332

[76] Liu B, Eltanahy EE, Liu H, Chua ET, Thomas-Hall SR, Wass TJ, Pan K, Schenk PM. Growth-promoting bacteria double eicosapentaenoic acid yield in microalgae. Bioresour Technol. 2020;316:123916. 32768998 10.1016/j.biortech.2020.123916

[77] Lin G, Sun F, Wang C, Zhang L, Zhang X. Assessment of the effect of Enteromorpha prolifera on bacterial community structures in aquaculture environment. PLoS ONE. 2017;12(7):e0179792. 28742878 10.1371/journal.pone.0179792 PMC5526538

[78] Ramadoss D, Lakkineni VK, Bose P, Ali S, Annapurna K. Mitigation of salt stress in wheat seedlings by halotolerant bacteria isolated from saline habitats. SpringerPlus. 2013;2(1):1–7. 23449812 10.1186/2193-1801-2-6 PMC3579424

[79] Pandey S, Gupta S. Diversity analysis of ACC deaminase producing bacteria associated with rhizosphere of coconut tree (Cocos nucifera L.) grown in Lakshadweep islands of India and their ability to promote plant growth under saline conditions. J Biotechnol. 2020;324:183–97. 33164860 10.1016/j.jbiotec.2020.10.024

[80] Bal HB, Nayak L, Das S, Adhya TK. Isolation of ACC deaminase producing PGPR from rice rhizosphere and evaluating their plant growth promoting activity under salt stress. Plant Soil. 2013;366(1–2):93–105.

[81] Etesami H, Alikhani HA. Suppression of the fungal pathogenMagnaporthe griseabyStenotrophomonas maltophilia, a seed-borne rice (Oryza sativaL.) Endophytic bacterium. Arch Agron Soil Sci. 2016;62(9):1271–84.

[82] Walitang DI, Kim K, Madhaiyan M, Kim YK, Kang Y, Sa T. Characterizing endophytic competence and plant growth promotion of bacterial endophytes inhabiting the seed endosphere of Rice. BMC Microbiol. 2017;17(1):209. 29073903 10.1186/s12866-017-1117-0 PMC5658939

[83] Sharma A, Shankhdhar D, Shankhdhar SC. Enhancing grain iron content of rice by the application of plant growth promoting rhizobacteria. Plant Soil Environ. 2013;59(2):89–94.

[84] Shekhawat K, Saad MM, Sheikh A, Mariappan K, Al-Mahmoudi H, Abdulhakim F, Eida AA, Jalal R, Masmoudi K, Hirt H. Root endophyte induced plant thermotolerance by constitutive chromatin modification at heat stress memory gene loci. EMBO Rep. 2021;22(3):e51049. 33426785 10.15252/embr.202051049 PMC7926228

[85] Andres-Barrao C, Alzubaidy H, Jalal R, Mariappan KG, de Zelicourt A, Bokhari A, Artyukh O, Alwutayd K, Rawat A, Shekhawat K et al. Coordinated bacterial and plant sulfur metabolism in Enterobacter sp. SA187-induced plant salt stress tolerance. *Proceedings of the National Academy of Sciences of the United States of America* 2021, 118(46). 10.1073/pnas.2107417118 PMC8609655 34772809

