# Supplementary material for: Unveiling the bacterial diversity and potential of the Avicennia marina ecosystem for enhancing plant resilience to saline conditions
Source: Environ Microbiome. 2024 Dec 4;19:101. doi: 10.1186/s40793-024-00642-w (PMC11619459; doi:10.1186/s40793-024-00642-w)
Supplement: Supplementary file 1 — Supplementary Material 1 [file 40793_2024_642_MOESM1_ESM.docx]

**Unveiling the bacterial diversity and potential of the *Avicennia marina* ecosystem for enhancing plant resilience to saline conditions**

**Amal Khalaf Alghamdi^1,2^, Sabiha Parween^1^, Heribert Hirt^1,3*^, and Maged M. Saad^1*^**

^1^DARWIN21, Biological and Environmental Science and Engineering Division, King Abdullah University of Science and Technology (KAUST), 23955-6900 Thuwal, Saudi Arabia

^2^Department of Botany and Microbiology, College of Science, King Saud University, P.O. Box 2455, Riyadh 11451, Saudi Arabia

^3^Max Perutz Laboratories, University of Vienna, Vienna, Austria

***Corresponding authors**

**Email:** [**Maged.saad@kaust.edu.sa**](mailto:Maged.saad@kaust.edu.sa) **:** [**https://orcid.org/0000-0002-5655-8674**](https://orcid.org/0000-0002-5655-8674)

**Email:** [**Heribert.hirt@kaust.edu.sa**](mailto:Heribert.hirt@kaust.edu.sa)**, https://orcid.org/0000-0003-3119-9633**

Supplementary tables

Table 1S: Physicochemical properties and elemental composition of soil and plants compartments (BS: Bulk soil, RS: Rhizosphere, RP: Rhizoplane, R: Root endosphere, and L: Leaves endosphere). NA, Not applicable, ND, Not detected.

|  | **BS** | **RS** | **RP** | **R** | **L** |
| --- | --- | --- | --- | --- | --- |
| **Max. temperature (°C)** | 35 | NA | NA | NA | NA |
| **Average temperature (°C)** | 30 | NA | NA | NA | NA |
| **Min. temperature (°C)** | 25 | NA | NA | NA | NA |
| **Color and texture** | Brownish grey | NA | NA | Soft pale nude, black spots | Grayish green,firm tissue covered with glandular dots. |
| **pH** | 7.19 | NA | NA | NA | NA |
| **Moisture content (%)** | 21.5 | NA | NA | NA | NA |
| **Macronutrients** |  |  |  |  |  |
| **P (mg Kg^-1^)** | 2.59E2 | 4.11E2 | 3.17E2 | 1.91E3 | 9.44E2 |
| **K (mg Kg^-1^)** | 2.05E3 | 8.55E2 | 6.13E2 | 1.36E4 | 5.23E3 |
| **Ca (mg Kg^-1^)** | 1.79E5 | 51.83 | 7.02 | 2.87E3 | 1.12E4 |
| **Mg (mg Kg^-1^)** | 1.11E4 | 1.72E2 | 24.77 | 5.48E3 | 5.94E3 |
| **Na (mg Kg^-1^)** | 1.13E4 | 5.17 E3 | 3.25E3 | 3.79 E4 | 5.76E4 |
| **S (%)** | 1.60 | 1.78 | 1.84 | 2.64 | 2.35 |
| **N (%)** | 0.17 | 0.21 | 0.22 | 1.47 | 0.96 |
| **C (%)** | 8.05 | 3.07 | 2.63 | 32.20 | 29.26 |
| **H (%)** | 0.42 | 0.51 | 0.48 | 4.20 | 4.05 |
| **O%** | 12.10 | 3.96 | 3.29 | 19.90 | 20.09 |
| **Micronutrients** |  |  |  |  |  |
| **B (mg Kg^-1^)** | 43.88 | 0.96 | 0.27 | 46.49 | 37.23 |
| **Cu (mg Kg^-1^)** | 7.32 | 0.22 | 0.16 | 4.11 | 4.54 |
| **Fe (mg Kg^-1^)** | 1.10E4 | 1.52 | 1.29 | 2.85E3 | 1.35E3 |
| **Mn (mg Kg^-1^)** | 1.42E2 | 0.06 | 0.14 | 23.50 | 32.15 |
| **Zn (mg Kg^-1^)** | 30.51 | 0.19 | 0.02 | 47.13 | 45.36 |
| **Trace Elements** |  |  |  |  |  |
| **Al (mg Kg^-1^)** | 9.33E3 | 4.63 | ND | 1.55E2 | 1.09E3 |
| **Ba (mg Kg^-1^)** | 19.39 | 0.04 | ND | 0.83 | 3.16 |
| **Ce (mg Kg^-1^)** | 9.80 | 0.13 | 0.04 | 4.11 | 4.13 |
| **Cr (mg Kg^-1^)** | 34.22 | 0.02 | 0.02 | 24.41 | 4.71 |
| **Ni (mg Kg^-1^)** | 7.76 | ND | ND | 20.52 | 4.35 |
| **Sr (mg Kg^-1^)** | 2.10E3 | 0.77 | 0.14 | 66.69 | 131.77 |
| **Ti (mg Kg^-1^)** | 3.60E6 | 3.00E3 | ND | 2.20E5 | 1.50E6 |
| **V (mg Kg^-1^)** | 18.40 | 0.25 | ND | 8.22 | 4.32 |
| **As (mg Kg^-1^)** | 6.40 | ND | ND | 17.44 | ND |
| **Cd (mg Kg^-1^)** | ND | ND | ND | ND | ND |
| **Ag (mg Kg^-1^)** | 7.02 | ND | ND | 45.87 | 12.48 |
| **Co (mg Kg^-1^)** | 2.91 | ND | ND | ND | 0.46 |
| **Li (mg Kg^-1^)** | 3.49 | 0.02 | ND | 0.32 | 1.33 |

Table 2S: Summary of Tags for each sample.

| ***Sample Name*** | ***Raw PE(#)*** | ***Combind (#)*** | ***Qualified (#)*** | ***Nochime (#)*** | ***Base (nt)*** | ***AvgLen (nt)*** | ***Q20*** | ***Q30*** | ***GC%*** | ***Effective%*** |
| --- | --- | --- | --- | --- | --- | --- | --- | --- | --- | --- |
| ***BS1*** | 159,122 | 148,359 | 147,564 | 112,718 | 42,549,815 | 377 | 98.56 | 94.84 | 54.89 | 70.84 |
| ***BS2*** | 164,361 | 153,276 | 152,137 | 103,026 | 38,927,919 | 378 | 98.45 | 94.35 | 56.11 | 62.68 |
| ***BS3*** | 163,861 | 152,554 | 151,640 | 100,566 | 37,993,180 | 378 | 98.53 | 94.85 | 56.12 | 61.37 |
| ***BS4*** | 168,162 | 154,617 | 153,763 | 111,219 | 42,027,611 | 378 | 98.63 | 95.13 | 56.4 | 66.14 |
| ***BS5*** | 168,105 | 155,600 | 154,649 | 104,820 | 39,561,826 | 377 | 98.62 | 95.06 | 55.67 | 62.35 |
| ***RS1*** | 161,234 | 148,958 | 148,207 | 108,108 | 40,874,842 | 378 | 98.55 | 94.9 | 55.36 | 67.05 |
| ***RS2*** | 166,850 | 155,486 | 154,396 | 112,646 | 42,637,982 | 379 | 98.48 | 94.56 | 55.36 | 67.51 |
| ***RS3*** | 162,076 | 150,716 | 149,819 | 111,801 | 42,277,924 | 378 | 98.5 | 94.67 | 55.46 | 68.98 |
| ***RS4*** | 153,025 | 143,102 | 142,189 | 108,530 | 41,045,512 | 378 | 98.44 | 94.47 | 54.46 | 70.92 |
| ***RS5*** | 160,993 | 151,822 | 150,905 | 118,869 | 44,943,685 | 378 | 98.68 | 95.28 | 55.17 | 73.83 |
| ***RP1*** | 150,515 | 139,471 | 138,751 | 111,945 | 41,898,962 | 374 | 98.64 | 95.14 | 54.6 | 74.37 |
| ***RP2*** | 166,059 | 154,549 | 153,608 | 130,906 | 49,164,765 | 376 | 98.69 | 95.33 | 54.31 | 78.83 |
| ***RP3*** | 154,571 | 136,203 | 135,365 | 102,553 | 38,654,978 | 377 | 98.76 | 95.49 | 54.32 | 66.35 |
| ***RP4*** | 152,038 | 144,662 | 143,590 | 105,034 | 39,632,760 | 377 | 98.67 | 95.08 | 55.09 | 69.08 |
| ***RP5*** | 150,051 | 142,186 | 141,264 | 114,564 | 43,131,456 | 376 | 98.75 | 95.5 | 54.71 | 76.35 |
| ***ER1*** | 136,195 | 118,893 | 118,210 | 81,888 | 30,866,726 | 377 | 98.76 | 95.53 | 55.27 | 60.13 |
| ***ER2*** | 161,633 | 151,238 | 150,301 | 114,212 | 42,980,738 | 376 | 98.82 | 95.63 | 53.56 | 70.66 |
| ***ER3*** | 165,626 | 155,097 | 154,197 | 114,741 | 43,214,589 | 377 | 98.78 | 95.55 | 53.67 | 69.28 |
| ***ER4*** | 115,586 | 110,262 | 109,491 | 84,001 | 31,665,067 | 377 | 98.73 | 95.33 | 54.5 | 72.67 |
| ***ER5*** | 166,865 | 157,891 | 156,883 | 122,434 | 46,282,127 | 378 | 98.67 | 95.18 | 54.98 | 73.37 |
| ***EL1*** | 124,222 | 118,599 | 117,856 | 96,614 | 36,380,634 | 377 | 98.59 | 95.01 | 55.26 | 77.78 |
| ***EL2*** | 181,442 | 174,472 | 173,386 | 153,598 | 57,881,292 | 377 | 98.84 | 95.78 | 54.8 | 84.65 |
| ***EL3*** | 167,044 | 149,732 | 148,909 | 113,315 | 42,646,416 | 376 | 98.78 | 95.56 | 53.89 | 67.84 |
| ***EL4*** | 202,688 | 180,272 | 179,416 | 159,942 | 60,287,829 | 377 | 99.14 | 96.63 | 54.77 | 78.91 |
| ***EL5*** | 150,353 | 114,747 | 114,066 | 68,912 | 25,940,227 | 376 | 98.75 | 95.47 | 53.98 | 45.83 |

Raw PE, paired-end raw reads; Combined, original data were assembled by overlapped sequence; Qulified, low-grade quality and short-length reads were filtered out to generate qualified data; Nochime, chimera of qualified sequences were filtered out to generate effective sequences; Base, the base number of total data; AvgLen, average sequence length of qualified tags per reading; Q20, the percentage of bases with sequencing error rate <1%; Q30, the percentage of bases with sequencing error rate <0.1%; GC%, GC content and Effective %, the percentage of effective tags.

Table 3S: Estimated ASVs richness and diversity indices of the root-associated microbiome collected from Mangrove at KAUST.

| *Group* | *Diversity* | | *Richness* | | *Evenness* | *Coverage* |
| --- | --- | --- | --- | --- | --- | --- |
|  | ***Simpson*** | ***Shannon*** | ***Chao1*** | ***Observed_ASVs*** | ***Pielou_e*** | ***Goods_coverage*** |
| *Bulk Soil* | 0.9838  ±0.13a | 1.6623  ±0.743a | 3190.993  ±293.9a | 2650  ±255.69b | 0.84388  ±0.055a | 0.95148  ±0.0034a |
| *Rhizosphere* | 0.9752  ±0.022a | 9.4302  ±0.855a | 3006.6072  ±325.93a | 2519  ±287.73ab | 0.8338  ±0.055a | 0.9544  ±0.0043a |
| *Rhizoplane* | 0.9812  ±0.010a | 9.0956  ±0.387a | 2761.873  ±293.83a | 2295.2  ±219.07ab | 0.8158  ±0.064a | 0.9588  ±0.0066a |
| *Roots* | 0.9568  ±0.027a | 7.7912  ±0.494a | 2089.4098  ±132.74ab | 1669  ±118.41ac | 0.7272  ±0.039a | 0.966  ±0.0019a |
| *Leaves* | 0.9598  ±0.031a | 7.7532  ±0.767a | 1385.4542  ±227.16b | 1260.6  ±210.04c | 0.7566  ±0.066a | 0.9858  ±0.0044b |

Table 4S: ADONIS (permutational MANOVA or nonparametric MANOVA) of all the compartments.

| ***Vs_group*** | ***Df*** | ***Sums Of Sqs*** | ***Mean Sqs*** | ***F. Model*** | ***R2*** | ***Pr(>F)*** |
| --- | --- | --- | --- | --- | --- | --- |
| ***Endophytes-Rhizoplane*** | 1(8) | 0.42727(1.35593) | 0.42727(0.16949) | 2.5209 | 0.23961(0.76039) | 0.01 |
| ***Endophytes-Bulk soil*** | 1(8) | 0.63497(1.32179) | 0.63497(0.16522) | 3.8431 | 0.3245(0.6755) | 0.001 |
| ***Endophytes-Rhizosphere*** | 1(8) | 0.58691(1.51022) | 0.58691(0.18878) | 3.109 | 0.27986(0.72014) | 0.017 |
| ***Endophytes-Leaves*** | 1(8) | 0.3568(1.6360) | 0.3568(0.2045) | 1.7448 | 0.17905(0.82095) | 0.006 |
| ***Rhizoplane-Bulk soil*** | 1(8) | 0.60454(1.06557) | 0.60454(0.13320) | 4.5387 | 0.36198(0.63802) | 0.007 |
| ***Rhizoplane-Rhizosphere*** | 1(8) | 0.35219(1.25400) | 0.35219(0.15675) | 2.2468 | 0.21927(0.78073) | 0.018 |
| ***Rhizoplane-Leaves*** | 1(8) | 0.4125(1.3798) | 0.41250(0.17247) | 2.3918 | 0.23016(0.76984) | 0.026 |
| ***Bulk soil-Rhizosphere*** | 1(8) | 0.46506(1.21986) | 0.46506(0.15248) | 3.0499 | 0.27601(0.72399) | 0.006 |
| ***Bulk soil-Leaves*** | 1(8) | 0.65093(1.34562) | 0.65093(0.16820) | 3.8699 | 0.32603(0.67397) | 0.014 |
| ***Rhizosphere-Leaves*** | 1(8) | 0.6215(1.5340) | 0.62150(0.19176) | 3.2411 | 0.28833(0.71167) | 0.001 |

Df, degree of freedom; Sums Of Sqs, sums of squares of deviations; Mean Sqs, Sums Of Sqs/Df; F. Model, F-test value; R2, the explanation of grouping factor on difference of samples, calculated from the ratio of grouping variance and total variance. Pr ,P-value. Values in parentheses stands for Residual Error.

**Table 5S: Multi-response permutation procedure (MRPP) analysis among the five compartments.**

| ***Group*** | ***A*** | ***observed-delta**** | ***expected-delta**** | ***Significance*** |
| --- | --- | --- | --- | --- |
| ***Bulk soil-Leaves*** | 0.1451 | 0.5539 | 0.6479 | 0.011 |
| ***Bulk soil-Endophytes*** | 0.1487 | 0.5459 | 0.6413 | 0.01 |
| ***Endophytes-Leaves*** | 0.0482 | 0.623 | 0.6546 | 0.013 |
| ***Bulk soil-Rhizoplane*** | 0.1649 | 0.4964 | 0.5944 | 0.009 |
| ***Leaves-Rhizoplane*** | 0.07182 | 0.5735 | 0.6179 | 0.024 |
| ***Endophytes-Rhizoplane*** | 0.08499 | 0.5655 | 0.618 | 0.019 |
| ***Bulk soil-Rhizosphere*** | 0.1187 | 0.5234 | 0.5939 | 0.007 |
| ***Leaves-Rhizosphere*** | 0.1142 | 0.6005 | 0.678 | 0.007 |
| ***Endophytes-Rhizosphere*** | 0.1132 | 0.5925 | 0.6682 | 0.017 |
| ***Rhizoplane-Rhizosphere*** | 0.07343 | 0.543 | 0.586 | 0.034 |

The difference among groups is significant if the value in the column of Significance is less than 0.05.

*A small value in the column titled observe-delta indicates that the inner-group variation is small, while a large one in the column of expected-delta means that the inter-group variation is great.

Table 6S: AMOVA results.

| ***Vs_group*** | ***SS*** | ***df*** | ***MS*** | ***Fs*** | ***p-value*** |
| --- | --- | --- | --- | --- | --- |
| ***Endophytes-Leaves*** | 0.190656(0.793285) | 1(8) | 0.190656(0.0991606) | 1.92269 | 0.047 |
| ***Leaves-Rhizoplane*** | 0.446995(1.16362) | 1(8) | 0.446995(0.145453) | 3.07312 | 0.031 |
| ***Endophytes-Rhizosphere*** | 0.336412(0.700724) | 1(8) | 0.336412(0.0875905) | 3.84073 | 0.029 |
| ***Bulk soil-Rhizoplane*** | 0.454917(1.00115) | 1(8) | 0.454917(0.125143) | 3.63516 | 0.005 |
| ***Leaves-Rhizosphere*** | 0.338412(0.792712) | 1(8) | 0.338412(0.099089) | 3.41523 | 0.022 |
| ***Bulk soil-Rhizosphere*** | 0.134161(0.630237) | 1(8) | 0.134161(0.0787796) | 1.703 | 0.212 |
| ***Endophytes-Rhizoplane*** | 0.43132(1.07163) | 1(8) | 0.43132(0.133954) | 3.21991 | 0.032 |
| ***Rhizoplane-Rhizosphere*** | 0.3711(1.07106) | 1(8) | 0.3711(0.133883) | 2.77183 | 0.043 |
| ***Bulk soil-Endophytes-***  ***Leaves-Rhizoplane-Rhizosphere*** | 1.33394(2.14451) | 4(20) | 0.333486(0.107225) | 3.11014 | <0.001* |
| ***Bulk soil-Leaves*** | 0.343259(0.722798) | 1(8) | 0.343259(0.0903497) | 3.79922 | 0.04 |
| ***Bulk soil-Endophytes*** | 0.287627(0.630809) | 1(8) | 0.287627(0.0788512) | 3.64772 | 0.026 |

Note: Analysis of molecular variance (AMOVA) showing the partitioning of genetic variation within and between varieties (df = degree of freedom, SS = sum of squares, MS mean squares, Fs = F test value, P is based on 9999 permutations).

**Table 7S: Beneficial impact of the 40 bacterial isolates caused enhancement in FW**

| ***No.*** | ***Strain*** | ***Plant Assay*** | | ****Cel.*** | ****Sid.*** | ****P sol.*** | ****Pect.*** | ****Prot.*** | **IAA** | **score** |
| --- | --- | --- | --- | --- | --- | --- | --- | --- | --- | --- |
|  |  | ***½ MS*** | ***½ MS +100mM NaCl*** |  |  |  |  |  |  |  |
| *1* | ***AK001*** | 40.2 | 109.4 | + | + | - | + | - |  | 3 |
| *2* | ***AK002*** | 4.4 | 20.8 | - | + | - | - | + |  | 2 |
| *3* | ***AK004*** | -0.6 | 106.4 | - | + | - | + | - |  | 2 |
| *4* | ***AK013*** | 3.7 | -21.3 | - | + | - | - | + |  | 2 |
| *5* | ***AK024*** | 4 | 20.3 | - | - | - | + | + |  | 2 |
| *6* | ***AK031*** | 7.8 | 24.3 | + | + | - | + | + |  | 4 |
| *7* | ***AK042*** | -4.3 | 55.2 | - | + | - | + | - |  | 2 |
| *8* | ***AK054*** | -2.5 | -27.3 | - | - | - | + | + |  | 2 |
| *9* | ***AK056*** | 1.9 | 22.3 | - | + | - | + | - |  | 2 |
| *10* | ***AK057*** | 13.4 | 20.2 | - | + | - | + | - |  | 2 |
| *11* | ***AK073*** | 14.1 | 24.2 | - | + | - | + | + |  | 3 |
| *12* | ***AK076*** | 12.5 | 39.4 | - | + | - | + | - |  | 2 |
| *13* | ***AK086*** | 16.1 | 20.2 | - | + | - | + | + |  | 3 |
| *14* | ***AK103*** | 25.8 | 39.6 | - | + | - | + | - |  | 2 |
| *15* | ***AK109*** | 12.6 | 25.6 | + | + | - | + | - |  | 3 |
| *16* | ***AK116*** | 21.5 | 29.1 | - | + | - | + | - |  | 2 |
| *17* | ***AK118*** | -38.3 | -32 | - | + | - | + | + |  | 3 |
| *18* | ***AK119*** | -11 | -12.7 | + | + | - | - | + |  | 3 |
| *19* | ***AK121*** | -51.2 | -35.6 | + | + | - | + | + |  | 4 |
| *20* | ***AK122*** | -60.9 | 36.3 | - | + | - | + | - |  | 2 |
| *21* | ***AK123*** | 5.1 | 76 | - | + | - | - | - |  | 1 |
| *22* | ***AK124*** | 9.4 | -36.2 | + | - | - | + | - |  | 2 |
| *23* | ***AK129*** | 2.7 | 30.1 | + | - | - | + | + |  | 3 |
| *24* | ***AK130*** | 52.1 | 441.3 | + | - | - | + | - |  | 2 |
| *25* | ***AK132*** | -36.1 | -24.3 | - | + | - | + | - |  | 2 |
| *26* | ***AK140*** | -66.9 | 24.4 | + | + | - | + | + |  | 4 |
| *27* | ***AK141*** | -13.3 | -15.2 | - | + | - | + | + |  | 3 |
| *28* | ***AK144*** | 4.8 | 20.4 | - | + | + | + | - |  | 3 |
| *29* | ***AK146*** | -28 | -18.2 | + | - | - | + | + |  | 3 |
| *30* | ***AK150*** | 18.3 | 20.2 | - | + | - | + | - |  | 2 |
| *31* | ***AK151*** | -1.5 | 123.7 | - | + | - | + | - |  | 2 |
| *32* | ***AK154*** | 13.7 | 400 | - | + | - | + | - |  | 2 |
| *33* | ***AK157*** | 26.8 | 66.7 | - | + | - | + | - |  | 2 |
| *34* | ***AK164*** | 1.3 | 152.6 | - | + | - | - | - |  | 1 |
| *35* | ***AK171*** | 7.9 | 21 | - | + | - | + | - |  | 2 |
| *36* | ***AK179*** | 13.3 | 20.8 | + | + | - | + | + |  | 4 |

**of *A. thaliana* seedling in both normal (1/2MS) and stress conditions (1/2MS with 100mM NaCl).**

*Cell: cellulase production, Sid: siderophore production, P sol: Inorganic phosphate solubilization activity, Pect: Pectinase production, Prot: Protease production, IAA: indole- 3-acetic acid production, Score: the qualitative PGP traits results (-,+).

| ***No.*** | ***Strain*** | ***Plant Assay*** | | ***Cel.*** | ***Sid.*** | ***P sol.*** | ***Pect.*** | ***Prot.*** | **IAA** | **score** |
| --- | --- | --- | --- | --- | --- | --- | --- | --- | --- | --- |
|  |  | ***½ MS*** | ***½ MS +100mM NaCl*** |  |  |  |  |  |  |  |
| *37* | ***AK180*** | 3.5 | 23.7 | - | + | - | + | + |  | 3 |
| *38* | ***AK181*** | 2.5 | 68.2 | + | + | + | + | + |  | 5 |
| *39* | ***AK185*** | 24.9 | 22.9 | + | + | - | + | - |  | 3 |
| *40* | ***AK197*** | -10.1 | 57.9 | - | + | - | + | + |  | 3 |
| *41* | ***AK204*** | 14.8 | 20.1 | + |  | - | + | + |  | 3 |
| *42* | ***AK205*** | 15.2 | 28.5 | - | + | - | + | - |  | 2 |
| *43* | ***AK214*** | 19.8 | 3.3 | + | + | - | + | + |  | 4 |
| *44* | ***AK215*** | -21.2 | 40.3 | + | - | - | + | - |  | 2 |
| *45* | ***AK218*** | 6.2 | 194.7 | - | + | - | + | - |  | 2 |
| *46* | ***AK222*** | 18.8 | 20.9 | + | + | - | + | + |  | 4 |
| *47* | ***AK223*** | 8.9 | 25 | + | + | - | + | + |  | 4 |
| *48* | ***AK229*** | 5.7 | 20.1 | - | + | - | + | - |  | 2 |
| *49* | ***AK231*** | -5.5 | 119.2 | - | + | - | + | - |  | 2 |
| *50* | ***AK232*** | 27.7 | 25.4 | + | + | - | - | + |  | 3 |
| *51* | ***AK235*** | 1.1 | 159.1 | + | + | - | + | + |  | 4 |
| *52* | ***AK237*** | -5.4 | -51.8 | + | + | - | + | - |  | 3 |
| *53* | ***AK238*** | 2.0 | 20 | - | + | - | + | + |  | 3 |
| *54* | ***AK249*** | -6 | -74.5 | + | + | - | + | + |  | 4 |
| *55* | ***AK251*** | 18.4 | 27.3 | - | - | - | - | + |  | 1 |
| *56* | ***AK253*** | -25.9 | 12.6 | - | + | - | + | - |  | 2 |
| *57* | ***AK254*** | -37.9 | 84.5 | + | + | - | + | - |  | 3 |
| *58* | ***AK255*** | 62.9 | 452.2 | + | + | - | + | - |  | 3 |
| *59* | ***AK256*** | -16.2 | 12.6 | - | + | - | + | - |  | 2 |
| *60* | ***AK257*** | -1.5 | 40.3 | - | + | - | + | - |  | 2 |
| *61* | ***AK259*** | -20.7 | 10 | - | + | - | + | + |  | 3 |
| *62* | ***AK260*** | -12.2 | -26.2 | - | + | - | + | - |  | 2 |
| *63* | ***AK261*** | -48.8 | -30.3 | + | + | - | + | + |  | 4 |
| *64* | ***AK262*** | -37 | 29 | - | + | - | + | - |  | 2 |
| *65* | ***AK263*** | -51.2 | -30.4 | - | + | - | + | + |  | 3 |
| *66* | ***AK264*** | -6.6 | 13.1 | - | + | - | + | - |  | 2 |
| *67* | ***AK266*** | 3.3 | 59.7 | - | + | - | + | - |  | 2 |
| *68* | ***AK267*** | -12.4 | 57.8 | - | + | - | + | - |  | 2 |
| *69* | ***AK268*** | 68 | 86.1 | - | + | - | + | - |  | 2 |
| *70* | ***AK269*** | -30.7 | 97.3 | - | + | + | + | + |  | 4 |
| *71* | ***AK270*** | -6.8 | -35.1 | + | + | - | + | + |  | 4 |
| *72* | ***AK271*** | 36.5 | 53.4 | - | + | - | + | - |  | 2 |

**Continue_2: Table 7**

*Cell: cellulase production, Sid: siderophore production, P sol: Inorganic phosphate solubilization activity, Pect: Pectinase production, Prot: Protease production, IAA: indole- 3-acetic acid production, Score: the qualitative PGP traits results (-,+).

| ***No.*** | ***Strain*** | ***Plant Assay*** | | ***Cel.*** | ***Sid.*** | ***P sol.*** | ***Pect.*** | ***Prot.*** | **IAA** | **score** |
| --- | --- | --- | --- | --- | --- | --- | --- | --- | --- | --- |
|  |  | ***½ MS*** | ***½ MS +100mM NaCl*** |  |  |  |  |  |  |  |
| *73* | ***AK273*** | 4.6 | 207.2 | + | + | - | + | + |  | 4 |
| *74* | ***AK274*** | -12.4 | 65 | - | + | - | + | - |  | 2 |
| *75* | ***AK275*** | 39.2 | 88.5 | - | + | - | + | - |  | 2 |
| *76* | ***AK276*** | -33.3 | 101.9 | + | + | - | + | + |  | 4 |
| *77* | ***AK277*** | 1.7 | 152.4 | - | + | - | + | - |  | 2 |

**Continue_3: Table 7**

*Cell: cellulase production, Sid: siderophore production, P sol: Inorganic phosphate solubilization activity, Pect: Pectinase production, Prot: Protease production, IAA: indole- 3-acetic acid production, Score: the qualitative PGP traits results (-,+).
