# Supplementary material for: Unveiling the bacterial diversity and potential of the Avicennia marina ecosystem for enhancing plant resilience to saline conditions
Source: Environ Microbiome. 2024 Dec 4;19:101. doi: 10.1186/s40793-024-00642-w (PMC11619459; doi:10.1186/s40793-024-00642-w)
Supplement: Supplementary file 2 — Supplementary Material 2 [file 40793_2024_642_MOESM2_ESM.docx]

**Unveiling the bacterial diversity and potential of the *Avicennia marina* ecosystem for enhancing plant resilience to saline conditions**

**Amal Khalaf Alghamdi^1,2^, Sabiha Parween^1^, Heribert Hirt^1,3*^, and Maged M. Saad^1*^**

^1^DARWIN21, Biological and Environmental Science and Engineering Division, King Abdullah University of Science and Technology (KAUST), 23955-6900 Thuwal, Saudi Arabia

^2^Department of Botany and Microbiology, College of Science, King Saud University, P.O. Box 2455, Riyadh 11451, Saudi Arabia

^3^Max Perutz Laboratories, University of Vienna, Vienna, Austria

***Corresponding authors**

**Email:** [**Maged.saad@kaust.edu.sa**](mailto:Maged.saad@kaust.edu.sa) **:** [**https://orcid.org/0000-0002-5655-8674**](https://orcid.org/0000-0002-5655-8674)

**Email:** [**Heribert.hirt@kaust.edu.sa**](mailto:Heribert.hirt@kaust.edu.sa)**, https://orcid.org/0000-0003-3119-9633**

Supplement figures:

Figure 1S: Workflow for sample processing to extract genomic DNA for microbiome analysis.


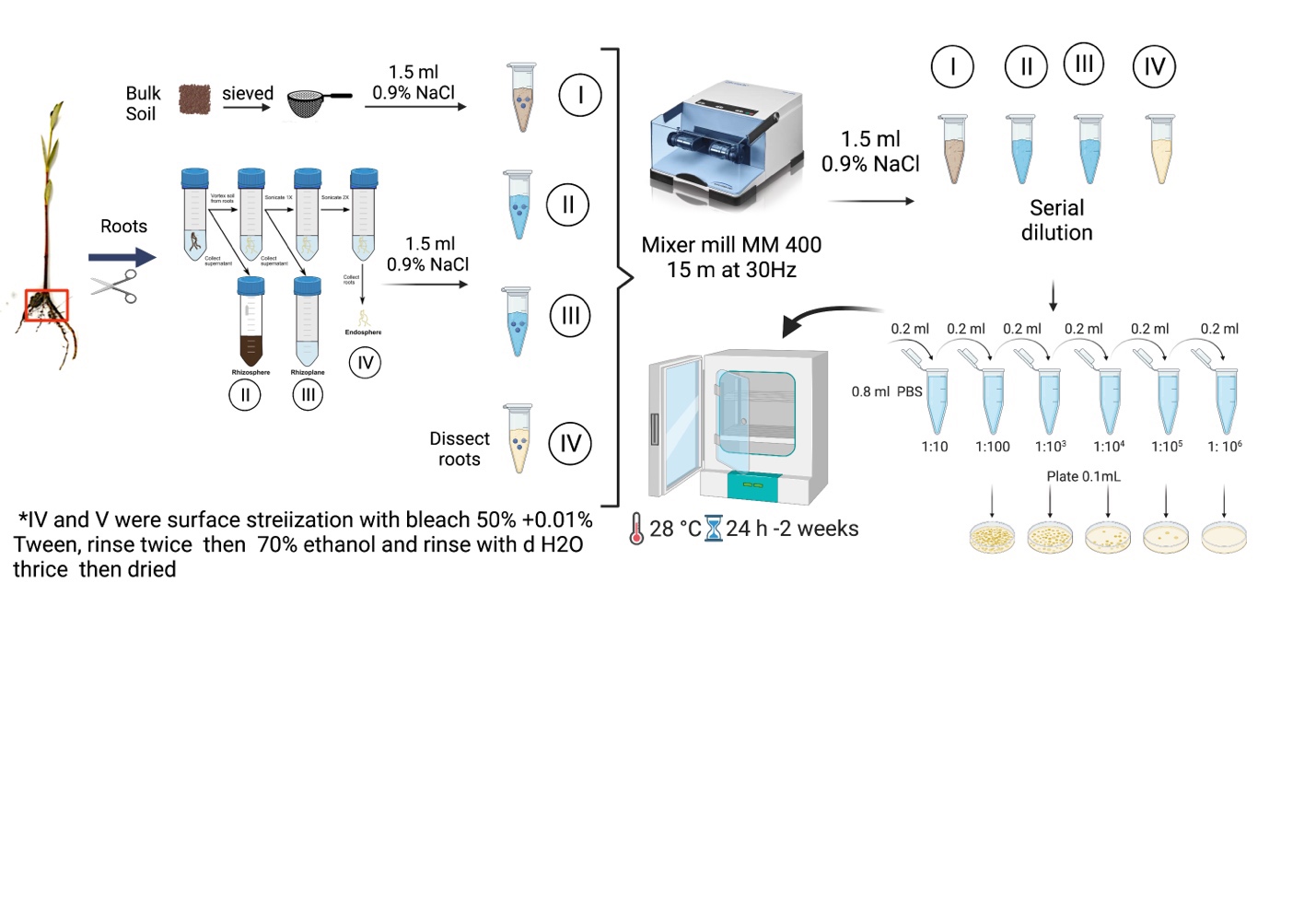


Figure 2S: Workflow of the culturally dependent steps during the isolation *Avicennia marina* bacterial collection of KAUST mangrove.


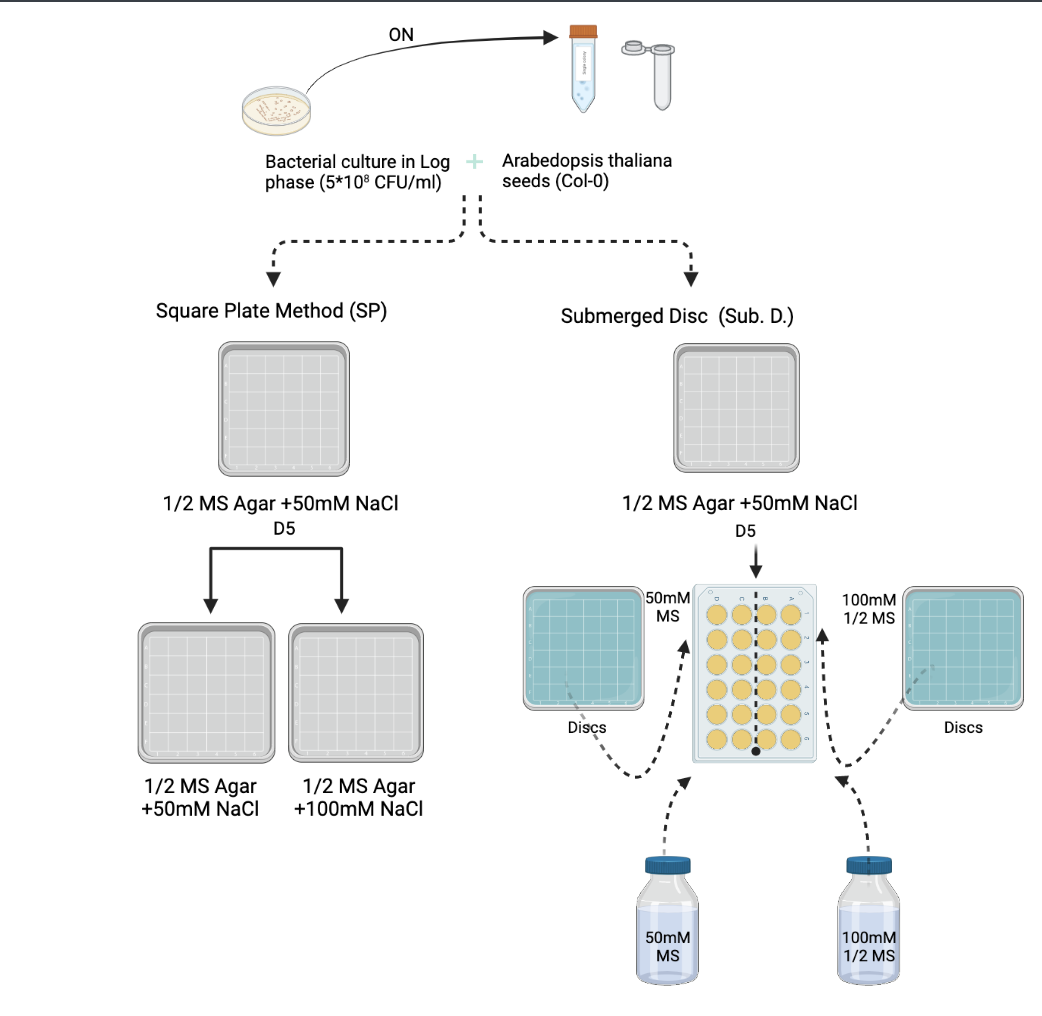


Figure 3S: The initial screening for the potential PGPB of the isolated mangrove’s bacterial collection using a model plant (*A. thaliana*).


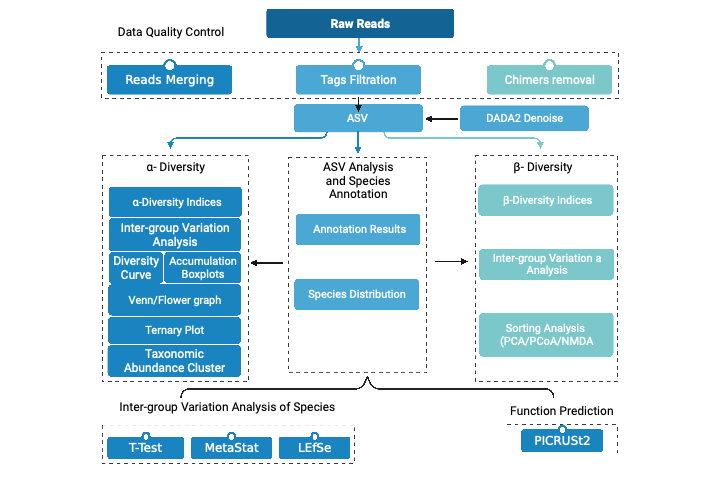


Figure 4S: Data analysis in the QIIME 2-2019.10  (version 1.7.0, [https://docs.qiime2.org](https://docs.qiime2.org/)) pipeline for studying *Avicennia marina*-associated microbiome in KAUST mangrove.


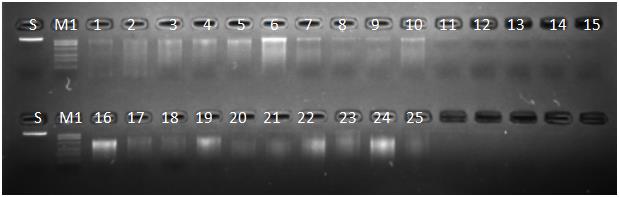


**Figure 5S: The genomic DNA extraction quality control (QC).** GelConc.：1% agarose

, Voltage：100v Run Time：40mi. S, standard sample (50ng); M1, Trans 15k plus (loaded 2μL); samples ranged in the order of upper table (All loaded 2μL).


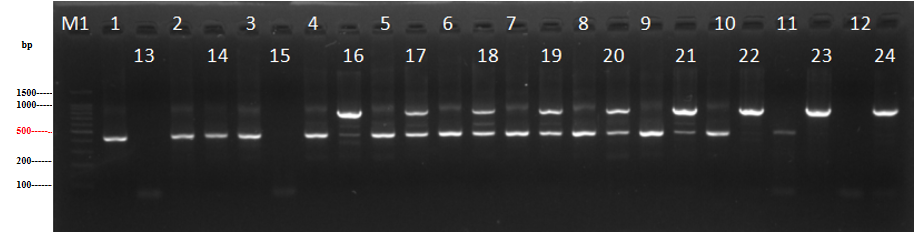

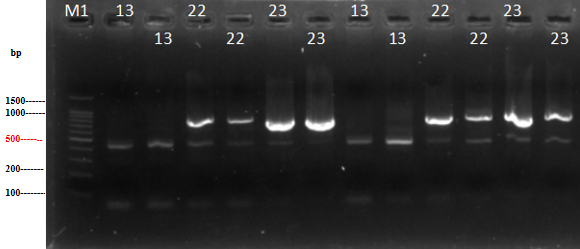


). **Figure 6S: The gel showing the PCR products using primers V5-V7.** GelConc.：2% agarose _Voltage 80v Run Time：40min. M1, Mol. Weight marker, 100bp ladder (loaded 1μL); samples ranged in the order of upper table (All loaded 3μL

A

B

**Figure 7S:** Comparative composition and relative abundance of the top taxa. A- The phyla. B- 13 bacterial genera associated with *A. marina* in bulk soil, rhizosphere, rhizoplane, roots endosphere, and leaves endosphere. The column's color represents the different compartments, and the length of the column represents the proportion size of the genus in each compartment.


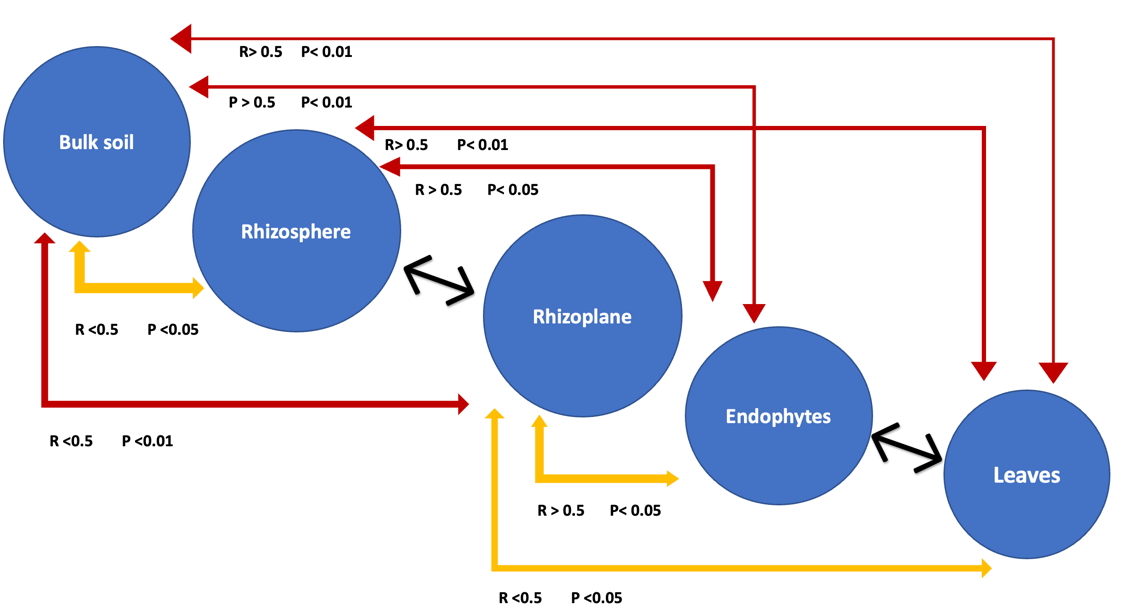


Figure S8: ANOSIM analysis.

R values that result from ANOSIM testing can be described as follows: 0.75 < R < 1, highly different; 0.5 < R < 0.75, different; 0.25 < R < 0.5, different with some overlap; 0.1 < R < 0.25, similar with some differences or high overlap; R < 0.1, similar.


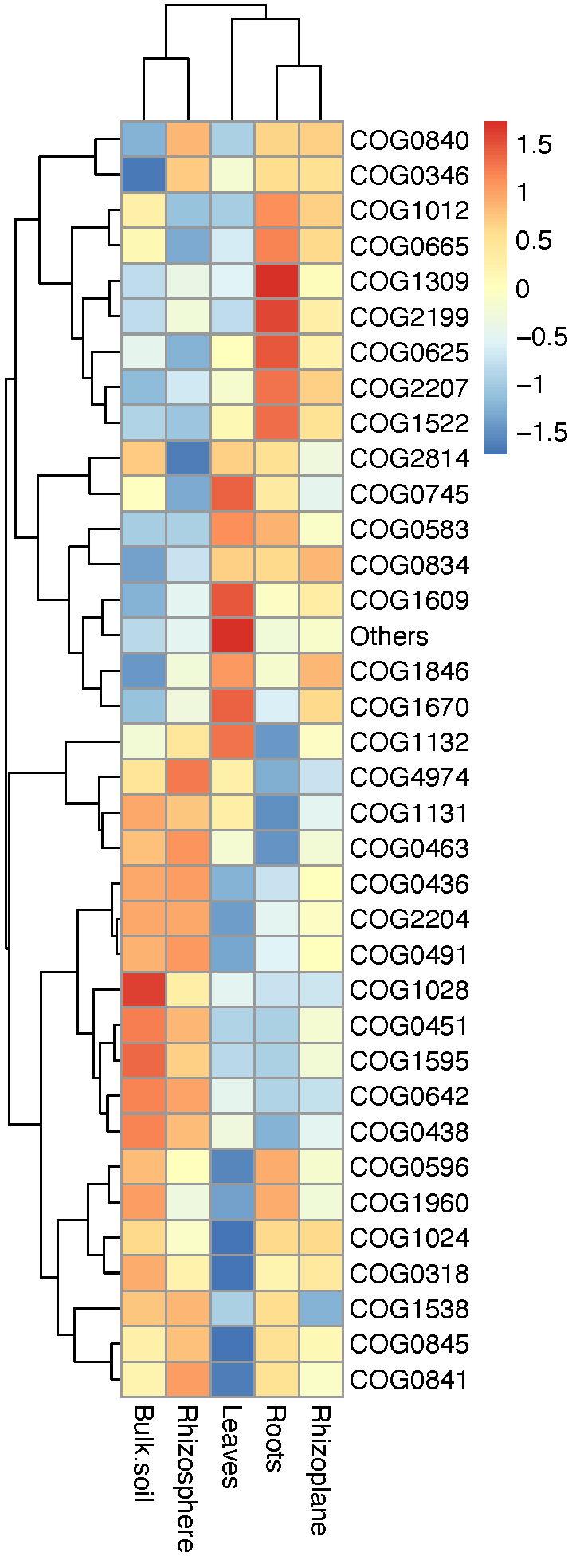


Figure 9S: Heatmap of the top 35COGs of the annotated Amplicon Sequence Variants (ASVs) across the five compartments of Mangrove.


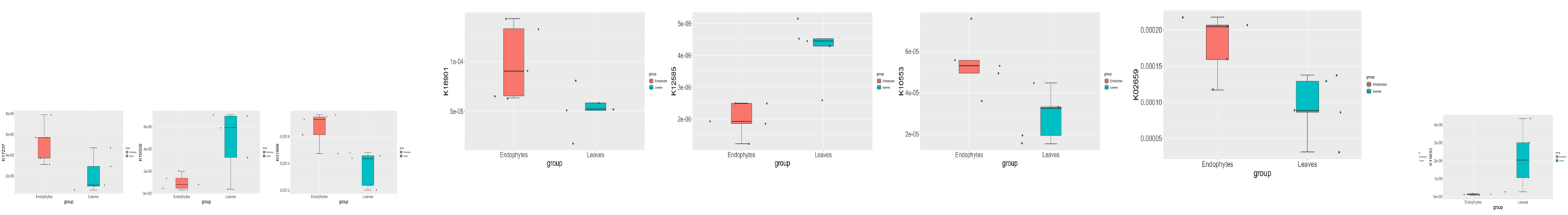

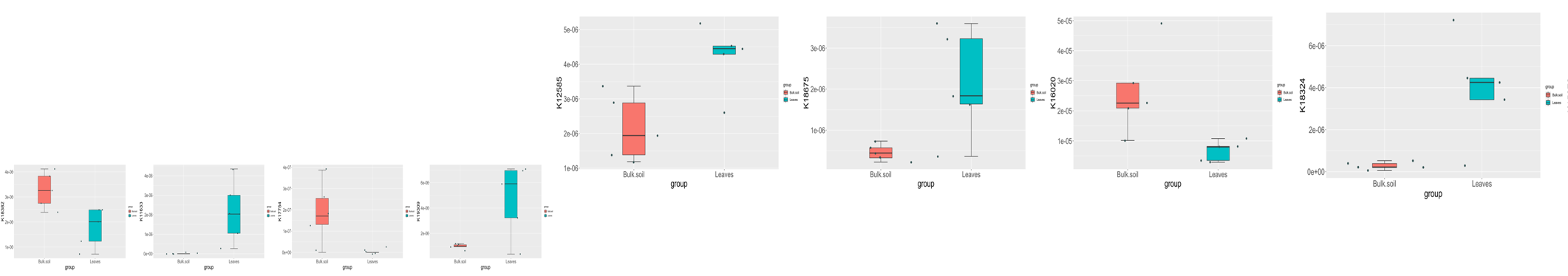

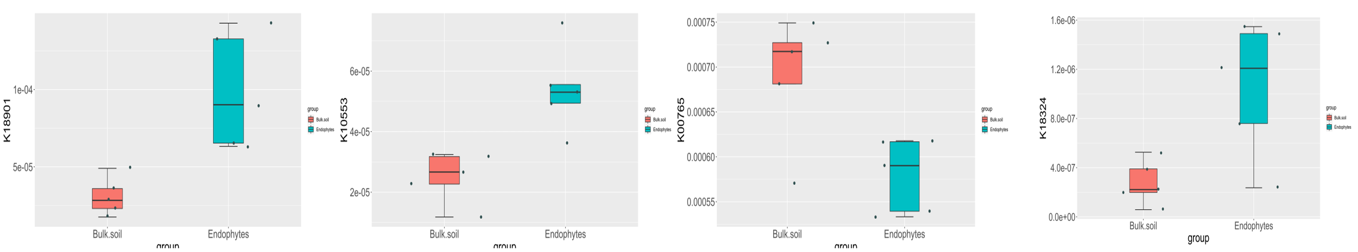

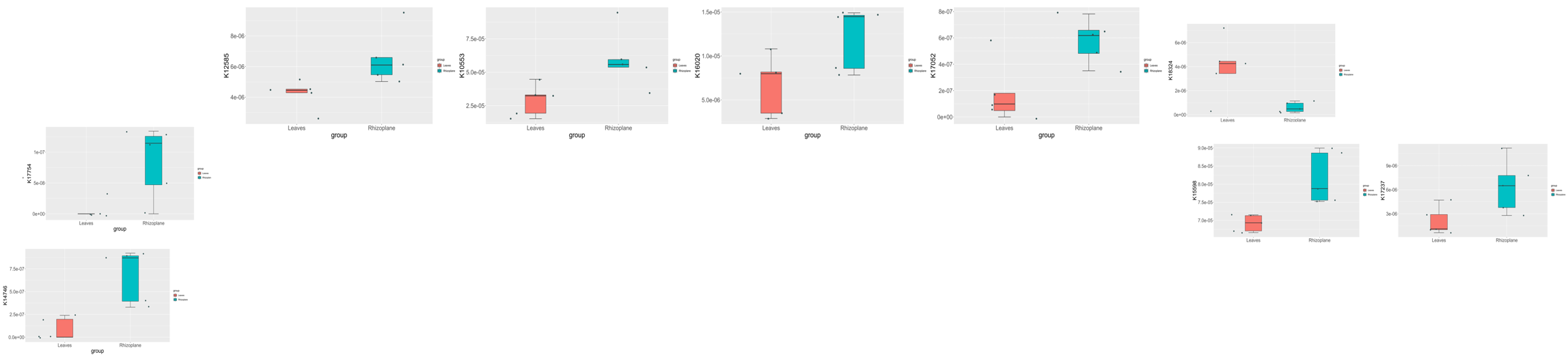

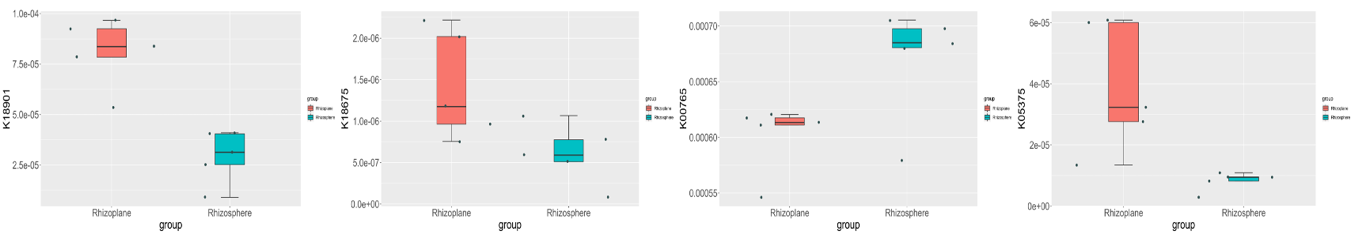

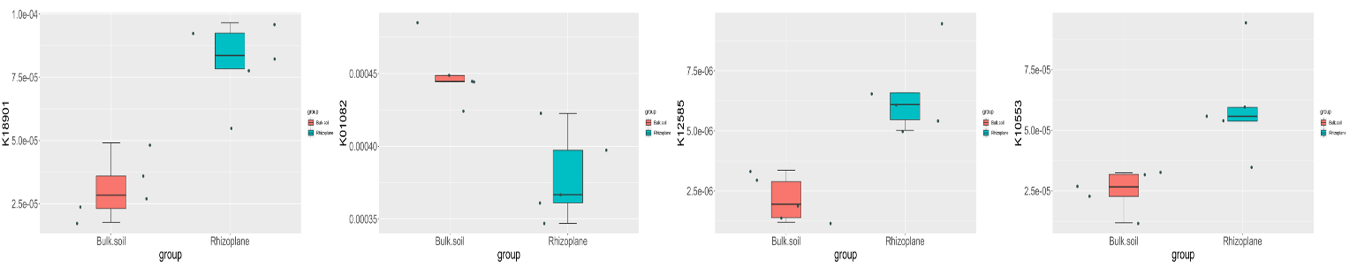

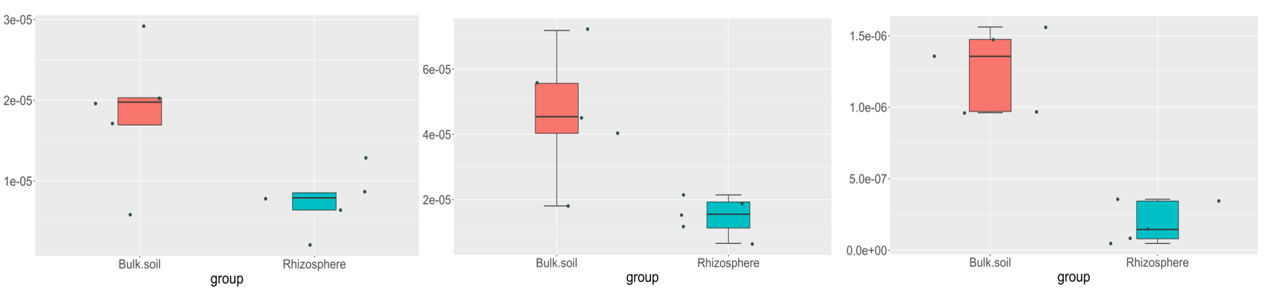


**G**

**F**

**E**

**D**

**C**

**B**

**A**

Figure 10S: The functional KO differences among the compartments. A- Roots vs. leaves, B- Soil vs. leaves, C- Soil vs. Roots, D- Rhizoplane vs. leaves, E- Rhizoplane vs. Rhizosphere, F- Soil vs. Rhizoplane, G- Soil vs. Rhizosphere.


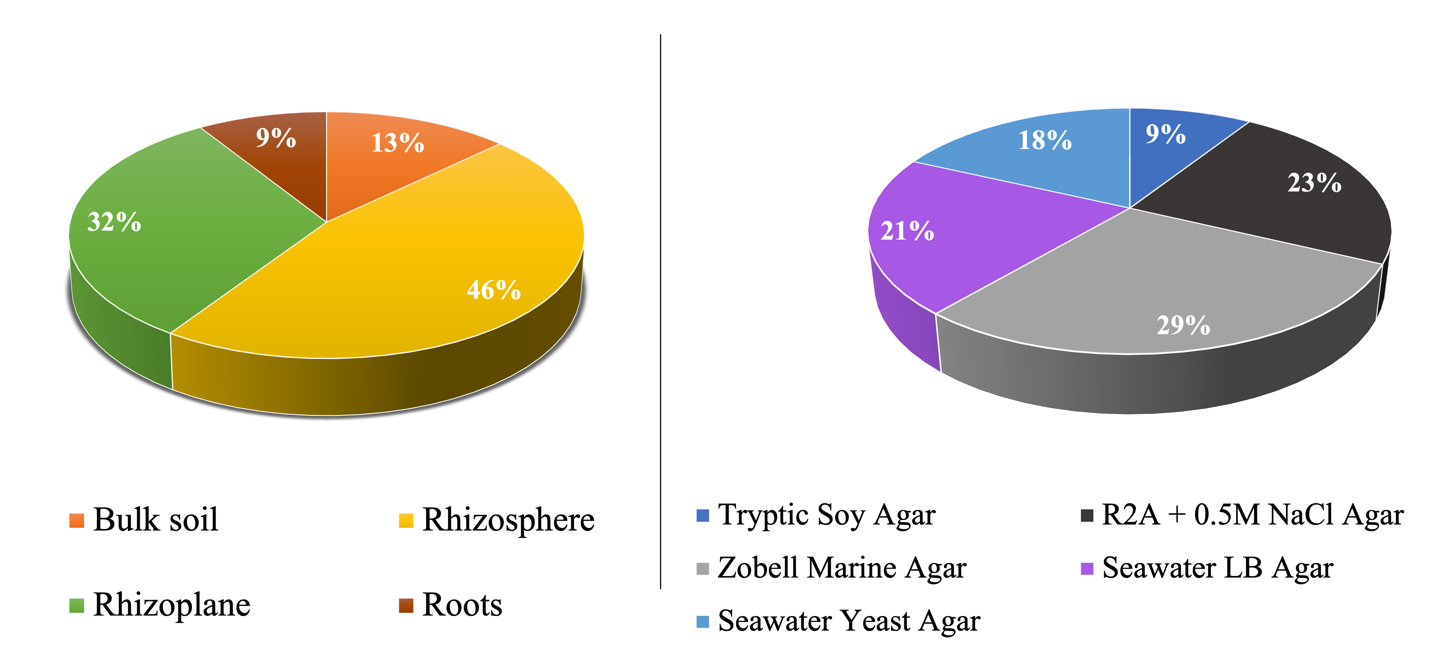


**B**

**A**

Figure 11S:A- The distribution of the bacterial isolates according to their compartment of origin. B- The distribution of bacterial Isolates on different culture media (A, Tryptic Soy Agar (TSA); B, R2A+0.5 M NaCl g/L; C, Zobell Marine Agar; D, SLB Agar; SY Agar).

**B**

**A**


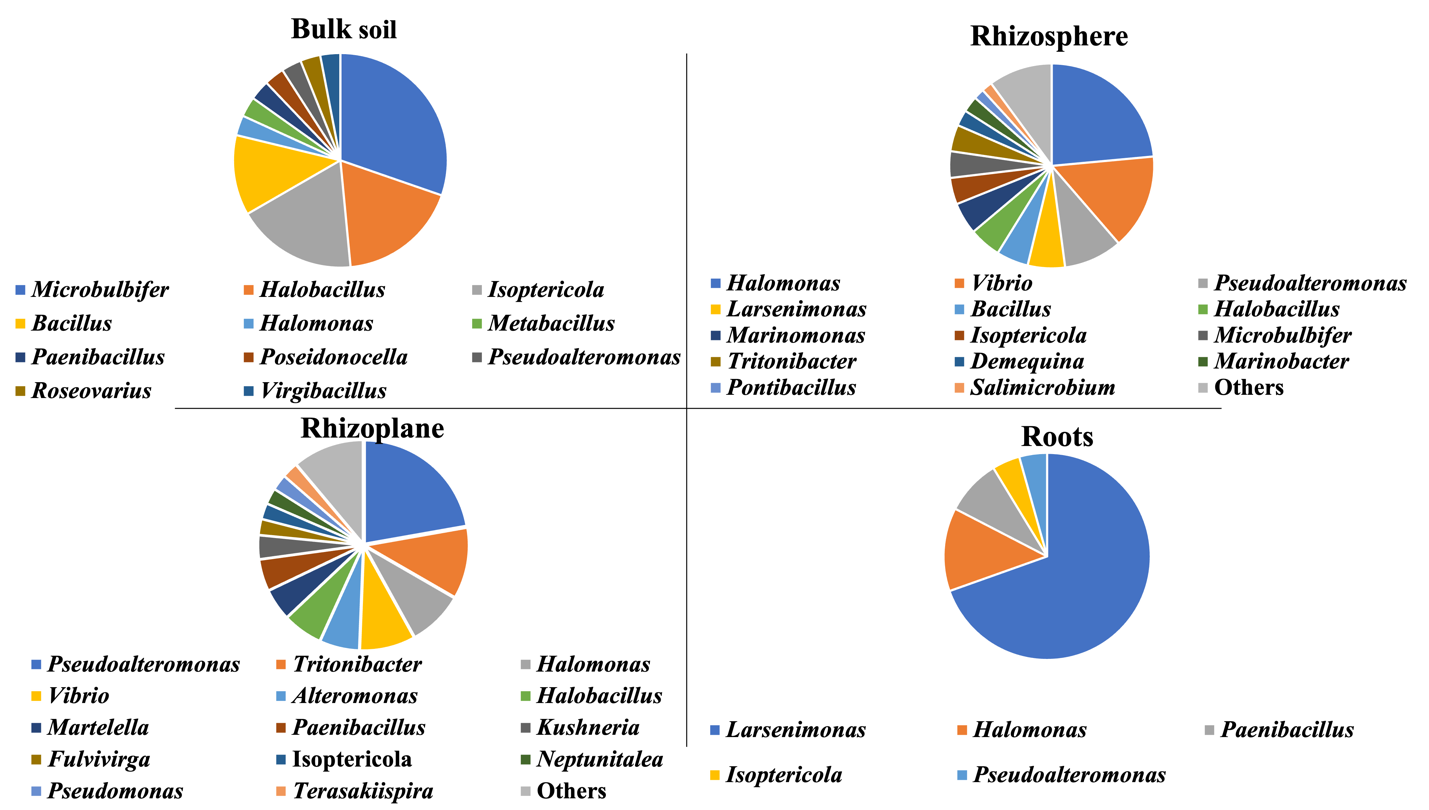


**C**

**D**

Figure 12S: A- The relative abundance of cultural genera in bulk soil was *Microbulbifer* (10%) followed by *Halobacillus* and *Isoptericola* (6%). B- The relative abundance of the genera in rhizosphere were *Halomonas* (28%) followed by *Vibrio* (18%). C- The abundant genera in the rhizoplane was *Pseudoalteromonas* (23%) followed by *Tritonibacter* (11%) and *Halomonas* (9%). D- The abundant genera in the root endophyte compartment was *Larsenimonas* (16.7%).

**
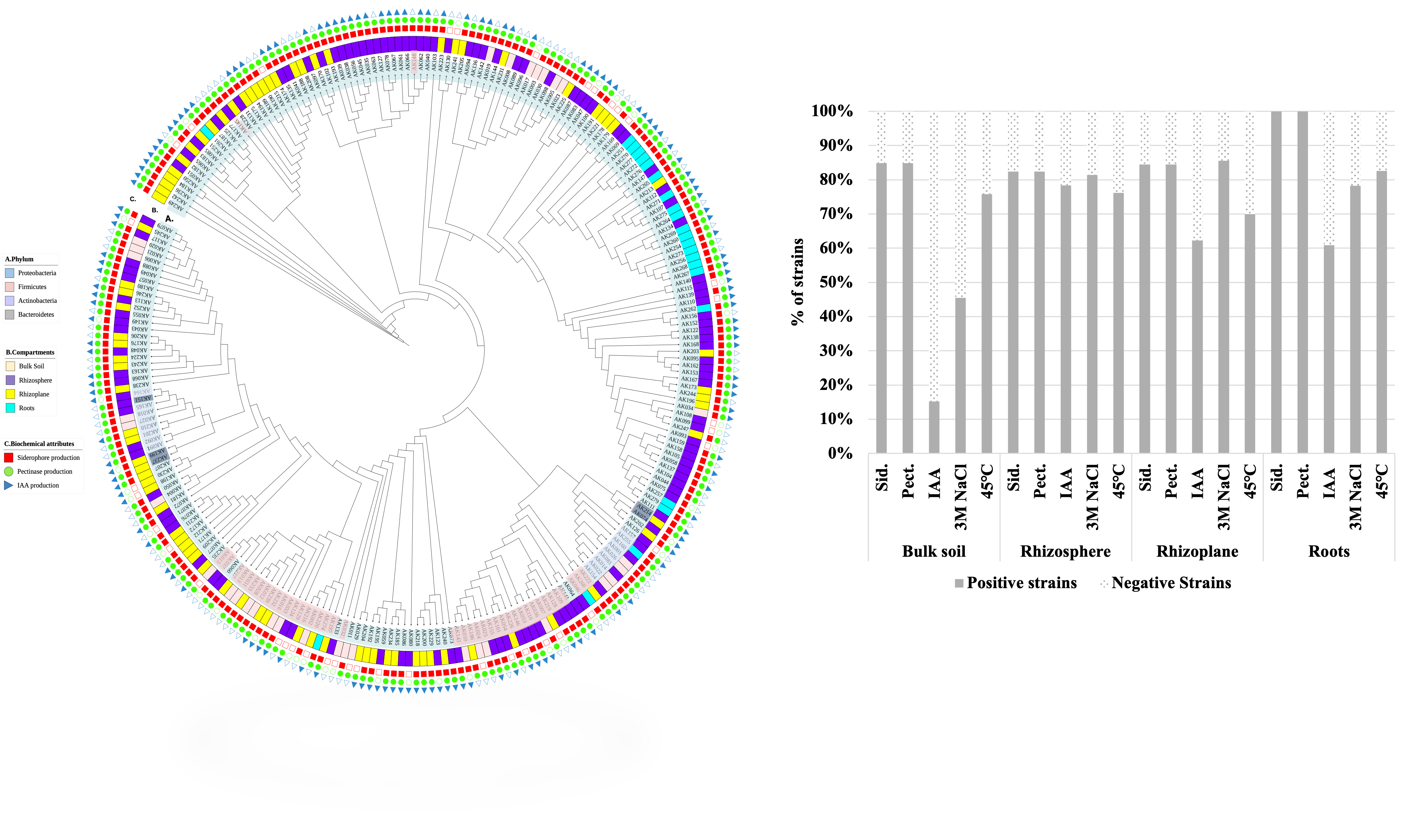
**

Figure 13S: AK Culture collection with PGP traits and stress tolerance to heat and salt stress.


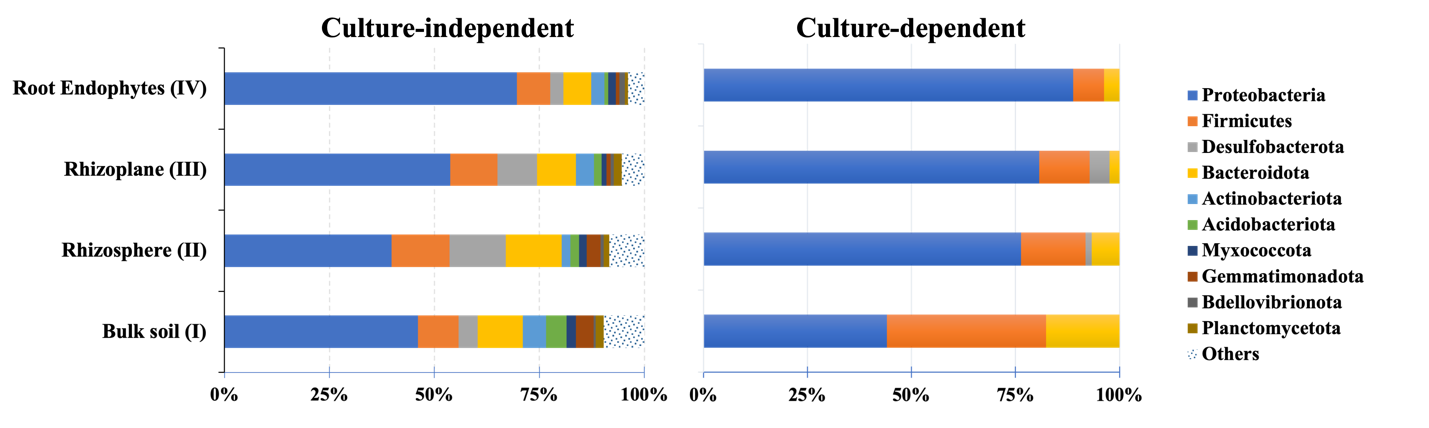


Figure 14S: The similarities and differences in relative abundance of the recovered genera in cultural-dependent analysis compared with the cultural-independent results. The difference at the level of Phyla.


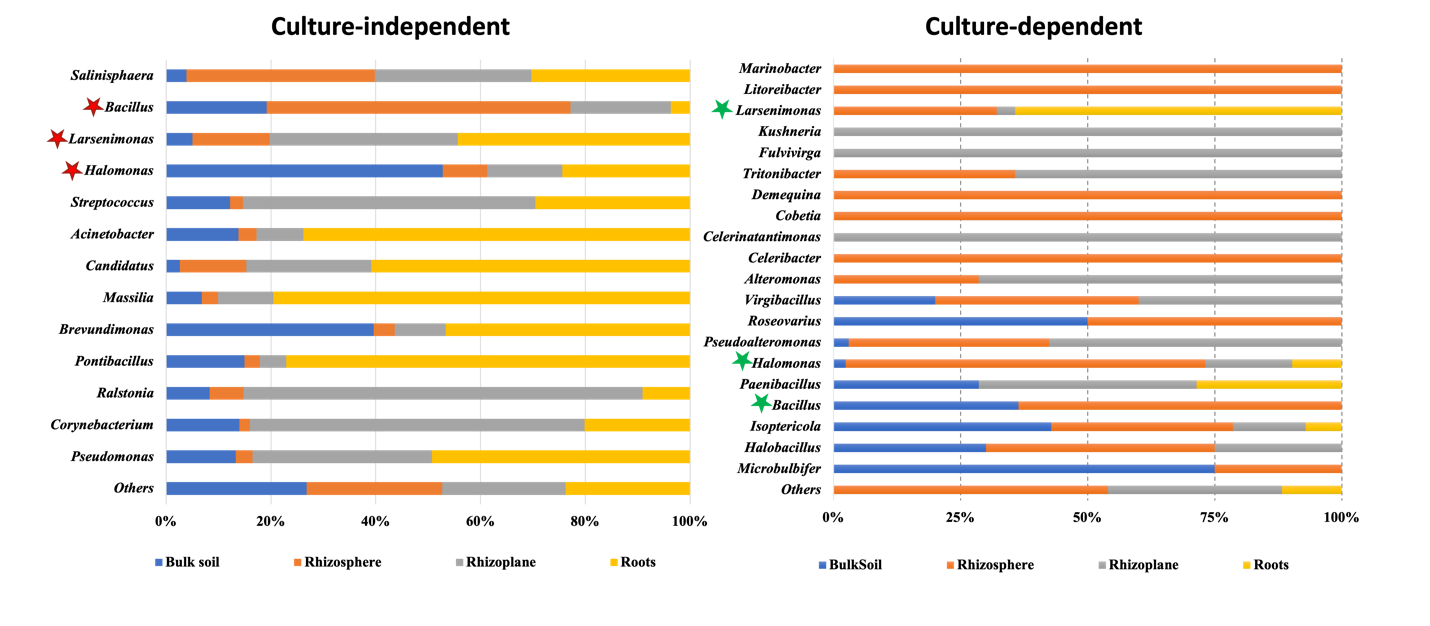
 Figure 15S: The similarities and differences in relative abundance of the recovered genera in cultural-dependent analysis compared with the cultural-independent results.

Figure 16S: Box plots of means of *A. thaliana* grown on 1/2MS and inoculated with the candidate bacteria in comparison with non-inoculated (mock) plants.

Figure 17S: Box plots of means of *A. thaliana* grown on 1/2MS + 100mM NaCl and inoculated with the candidate bacteria in comparison with non-inoculated (mock) plants.

Figure 18S: Box plots of means of *A. thaliana* grown on 1/2MS using SDM and inoculated with the candidate bacteria in comparison with non-inoculated (mock) plants.

Figure 19S: Box plots of means of *A. thaliana* grown on 1/2MS + 100mM NaCl using SDM and inoculated with the candidate bacteria in comparison with non-inoculated (mock) plants.


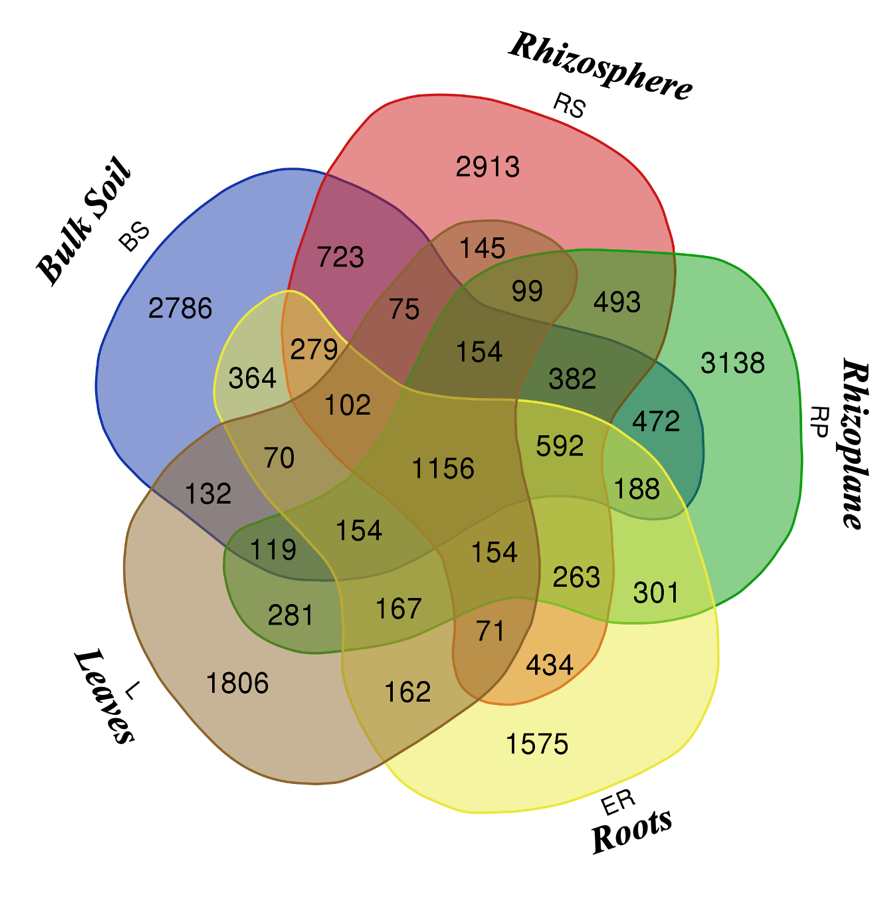


Figure 20S:The Vienn distribution of Amplicon Sequence Variants (ASVs) across the five compartments of Mangrove sp. The ASVs across compartments shown in different colors. The Bulk soil in total encodes for 7748 ; Rhizosphere (RS) 8035; Rhizoplane (RP) 8113; Roots (R ) 6032; and Leaves (L) 4847 ASVs.


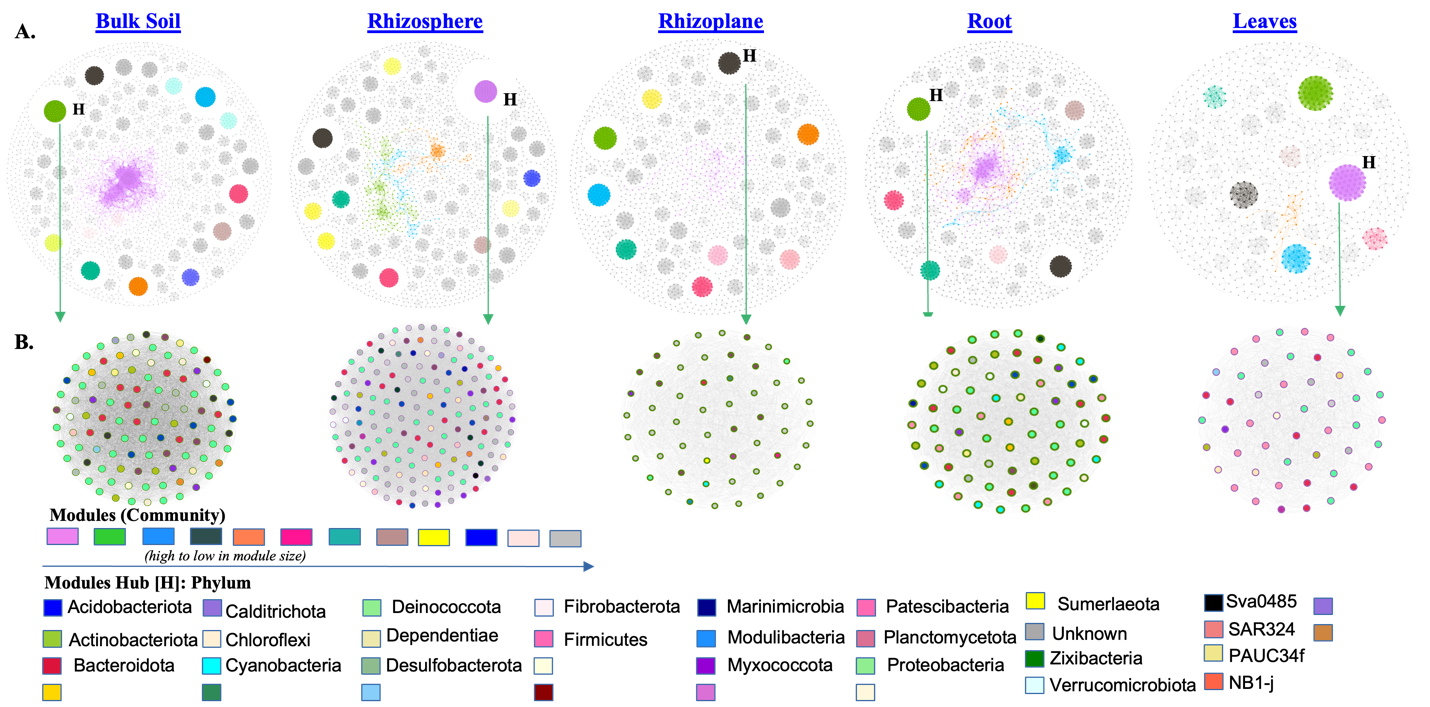


Figure 21S: The co-occurrence networks among taxa in different compartments of Mangrove sp. (A) The networks across each compartments where the solid color filled circles (nodes) represents taxa and connecting links (edges) stands for a strong (Spearman’s corr. |>=0.9|) and significant (P-value 0.01) correlation which clustered to different modules or communities. Only the modules with high numbers of nodes highlighted by distinct colors. Size cut-off >=30. (B) The hub ([H]:high degree of connectivity) modules of each compartments, colored by taxonomic affiliations at phylum level.
